# Visual Background Choice and Light Environment Affect Male Guppy Visual Contrast

**DOI:** 10.3390/vision6030056

**Published:** 2022-09-07

**Authors:** John A. Endler, Dara-Marie Raggay, Solomon Maerowitz-McMahan, David N. Reznick, Rebecca C. Fuller

**Affiliations:** 1 Centre for Integrative Ecology, School of Life and Environmental Sciences, Deakin University, Waurn Ponds, VIC 3216, Australia; 2 Department of Life Sciences, University of the West Indies, St Augustine 999183, Trinidad and Tobago; 3 Department of Biology, University of California, Riverside, CA 92521, USA; 4 School of Integrative Biology, University of Illinois at Urbana-Champaign, Champaign, IL 61820, USA

**Keywords:** mate choice, visual backgrounds, orientation, background choice, chromatic contrast, achromatic contrast

## Abstract

Male guppies (*Poecilia reticulata*) have multiple colored spots and perform courtship displays near the edges of streams in Trinidad in shallow water flowing through rainforest. Depending upon the orientation of the pair, the female sees the male displays against gravel or other stream bed substrates or against the spacelight—the roughly uniform light coming from the water column away from the bank. We observed courting pairs in two adjacent natural streams and noted the directions of each male display. We found that the female sees the male more often against spacelight than against gravel when females either faced the spacelight from the opposite bank or from downstream, or both. Visual modelling using natural substrate reflectances and field light measurements showed higher chromatic contrast of males against spacelight than against substrates independent of the two ambient light environments used during displays, but achromatic contrast depended upon the ambient light habitat. This suggests that courtship involves both chromatic and achromatic contrast. We conclude that the orientation of courting pairs and the ambient light spectrum should be accounted for in studies of mate choice, because the visual background and light affect visibility, and these differ with orientation.

## 1. Introduction

Female choice of males is known to be favored by higher chromatic and/or achromatic contrast within color patterns in a variety of species, both in the lab and the field (for example [1,2]). Male mating success is also favored by contrast with the visual background (examples include [1,2,3,4,5]). There is some evidence for habitat choice for specific light environments and/or visual backgrounds [3,4,6,7,8,9,10,11]. Choice of lighting or visual backgrounds can alter perception and mate choice [3,4]. Several species actually modify the visual backgrounds for increased contrast during courtship [7,12,13,14,15]. For example, *Lispe capa* fly males clasp and rotate a female such that she sees him with greater background contrast [16] and sticklebacks (*Gasterosteus aculeatus*) choose male nest depth, affecting visual backgrounds and lighting during mating, which affects both opsin gene regulation and visual contrast [1,17,18]. Here, we ask whether alterations in visual conditions occur simply through changes in orientation during courtship and whether these have meaningful effects on chromatic and/or luminance contrast in a species that does not actively manipulate visual backgrounds.

**Figure 1:**
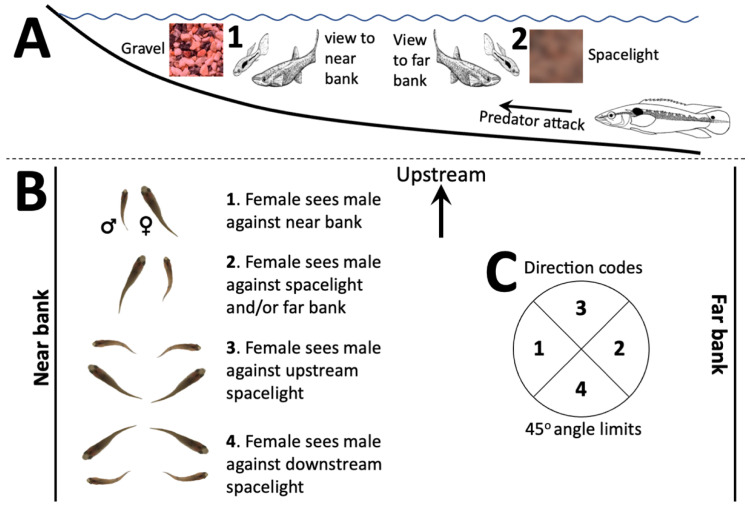
Guppy courtship location, orientation and orientation scoring scheme (**A**). Pairs court in the shallows and most predator attacks come from the depths. Near stream bank views tend to be gravel, rocks, dead leaves, and silt. Views in other directions consist mostly or entirely of sidewelling light (“spacelight”) (**B**). The courting pair (female larger) was scored as oriented in one of four directions relative to the near bank, relative to the female’s view of the male (**C**). A given direction code was recorded if the male’s orientation was within ±45° of the nominal direction. Guppy drawings in (**A**) are modified from [20].

Guppies live in rainforest streams in Venezuela and Trinidad and Tobago, and are a model system for exploring the evolutionary ecology and behavioral ecology of mate choice and the countervailing effects of predation [19]. Guppy courtship consists of a male approaching and displaying laterally (as in Figure 1) to a female 2–3 cm away, and mating occurs if the female is attracted enough for her to approach him [20]. During courtship, the female sees the male’s side, which is covered by colored patches [21]. If chosen, she will glide towards him. Guppies usually court in parts of streams with gently sloping banks (Figure 1), and courtship tends to occur at depths of about 5–20 cm, frequently over gravel. This means that most courtship occurs nearer to the stream bank than the depths (personal observations 1975–1991). Shallow water courtship may have evolved because predators are more common in deeper water and attacks come from relatively deeper water or from the direction of the opposite bank [21,22].

Courtship near the stream bank has the interesting consequence that visual backgrounds differ radically in spatial scale and color contrast depending upon gaze direction (Figure 1A). There are two reasons for this: visual acuity and water optics. Guppy visual acuity is about four cycles per degree from both anatomical and behavioral measurements [23,24]. Guppy males court females at 2–3 cm distance, and the sigmoid display (intense courtship stage in which his body forms an S-shape [20]) occurs at 2 cm or less [25]. This means that female guppies can see male spots of at least 0.2 mm initially and 0.1–0.14 mm diameter during sigmoid displays—essentially all male markings at ordinary courtship distance. Courting males are in front of gravel 10–20 cm from the female (mostly around 10 cm), so gravel above 0.67–1.33 mm will be seen during the displays and will affect male contrast. At 50, 100 and 200 cm, objects will be distinct if greater than 3.35, 6.67 or 13.3 mm in diameter, respectively. However, given the accommodation limits of small eyes like guppies’ [26], spatial resolution at longer distances is likely to be worse than predicted from retina and eye geometry. Acuity effects reduce both luminance and color contrast at these distances, making distant visual background patterns much less distinct [27]. In addition to acuity limits, the water contains tannins (“tea-stained”) and other materials which scatter and diffuse light. The resulting sidewelling light coming directly from the water column (called “spacelight”) will further blur the distant visual backgrounds and color mix them [28]. These effects will occur regardless of ambient light intensity. We will refer to the combined effects of visual acuity limits and spacelight simply as “spacelight” for brevity.

If the male is between the bank and the female, she will see him against the sloping gravel background; but if the female is between the bank and a male, she will see him against the spacelight (Figure 1A,B). Spacelight is likely to be spatially uniform or with weak gradients [28], whereas close gravel backgrounds should have a much higher variance in reflectance, color, and grain size [21,22] because they are in focus and close enough that water optics have little effect. This is likely to result in very different visual contrast between the male and the visual background, as seen by the female, depending upon the pair’s orientation.

We address two questions in this paper using wild guppies (*Poecilia reticulata*): (1) Do courting guppies prefer a particular orientation relative to the nearest stream bank? (2) If so, what are the male visual contrast consequences in the preferred and non-preferred directions?

## 2. Materials and Methods

### 2.1. Orientation during Courtship

We observed guppy courtship in the Caigual (61.275° W 10.715° N) and Taylor (61.271° W 10.708° N) tributaries of the Guanapo river in the northern range of Trinidad, which have elevations of 297 and 281 m, respectively. These are shallow streams in rainforest less than 1 km apart and are typical of guppy populations at higher elevations in the Northern Range Mountains [29]. Locations and sample sites were chosen with habitats and microhabitats as similar as possible, shaded at the time of observations, as well as a locally higher guppy density to ensure good sample sizes. Observations were made while quietly sitting on the bank with no disturbance to the water or guppies. We did not investigate whether guppies chose locations with respect to light conditions, although we did record relative use of the different light environments during observations.

We collected courtship data from as many different males as possible, limited by population size. We sampled two adjacent streams to increase the sample size. As courtship is much less frequent in the middle of the day, [30] we recorded courtship behavior between 7–10 a.m. and 2–6 p.m. We observed the courtship of a focal male towards multiple females until we lost track of him; individual recognition was possible because male patterns are unique [21,22].

The recordings consisted of behavior sequences and light environment codes. Each time the focal male presented a sigmoid display to a female, we recorded the orientation of the pair (Figure 1) by means of an imaginary disk with four segments placed over the pair, with codes 1 to 4 indicating the position relative to the bank (Figure 1B,C). The four codes refer to the female’s view (eye direction), not the direction of her long axis (Figure 1B): *D1*, female views male against the near bank/gravel; *D2*, female views male against the spacelight (water column) and/or far bank; *D3*, female views male against upstream spacelight; *D4*, female views male against the downstream spacelight. A direction code was recorded for a focal male whenever we saw a sigmoid display. The display was assigned one of the four codes if within ±45° of the nominal direction of 180°, 0°, 90°, 270°, for codes *D1*, *D2*, *D3*, and *D4*, respectively, where 0° indicates females looking away from the adjacent stream bank, and angles measured counterclockwise (Figure 1C). In addition, code *LI* was recorded when the male lost interest in the female and swam away, *Lost* was recorded when we lost sight of the male and *C* was recorded when copulation occurred. For analysis, for each male we used the modal (most common) direction code, to avoid pseudoreplication, because males differed in the number of displays in their observed display sequences. When the most common direction codes were tied, we used the last tied code in the sequence.

In addition to the direction codes, data for that male also included an arbitrary male number, start time and light environment (see [31]). Our light categories reflect the forest canopy cover, cloud cover, and time of day. *FS* was coded when the pair was in shade when the sun was not blocked by a cloud; this is equivalent to Forest Shade in [31]. *SG* was coded when the pair was in a sun patch (sun not blocked by cloud); this is spectrally equivalent to small gaps [31] because the canopy gaps were small. *OC* was coded when the sun was blocked by a cloud; this is equivalent to Open/Cloudy (*OC*). All areas under a forest canopy converge on the same spectral shape when the sun is blocked by clouds, and they have the same spectral shape as sunlight from a large gap or in the open [31]. *EL* was coded for pairs active early in the morning and is equivalent to Early/Late (*EL*). See [31] for light environment details.

As pair orientation angles were discontinuous (recorded as one of 4 values), we could not use classical circular statistics; our data violate von Mises’s angle distribution assumption [32]. Consequently, we used permutation tests [33] with 200,000 permutations. The tests worked as follows: (1) The modal orientation angles of each fish were divided into the two groups to be tested, for example fish in the two streams or fish with short and long displays. (2) The circular mean modal vector was calculated for each of the two groups. (3) The difference between these group means was calculated; this is the observed value. (4) The group membership codes were permuted 200,000 times, leading to 200,000 mean vector differences. (5) The distribution of permuted differences was compared to the observed difference to get the probability of getting the observed value or a more extreme difference. We used a one-tailed test if we had an a priori mechanistic hypothesis about the sign of the difference and a two-tailed test if we had no mechanistic hypothesis.

### 2.2. Light Measurements

We collected substrate reflectance spectra (for near bank visual backgrounds), spacelight radiance (for far bank direction backgrounds), and both vertical and horizontal irradiance (for chromatic adaptation, substrate radiance calculations, and downward attenuation spectra) in multiple locations in both streams. We used Ocean Optics^®^ USB2000 and USB4000 spectrometers separately for irradiance and radiance. Radiance is the light coming from a small solid angle and irradiance is the light coming from 180° solid angle [34]. The UV-VIS fiber optic cables ended in a cosine-corrected tip for irradiance and a Gershun tube set for a 1° acceptance angle for radiance, both by Ocean Optics. Both systems were calibrated with a deuterium-halogen lamp. Spacelight is light coming from the water column [28] and was measured as radiance with the Gershun tube facing horizontally at various depths where guppies court. We calculated the downward attenuation coefficient (*K_d_*) by regressing downwelling irradiance log(intensity) on depth [35] for each measured wavelength to yield the attenuation spectrum. We calculated radiance for each guppy color patch and substrate component by multiplying its reflectance spectrum by the downward irradiance spectrum separately for *OC* and *FS*. For detailed methods see [34].

### 2.3. Guppy Color Patch Reflectance Spectra

We used our existing data [24] for the mean reflectance of all 13 color patch classes of 120 male guppies in the calculations. We were specifically interested in the contrast between these patches and each patch in the natural Trinidad stream bed substrates and the spacelight, and not the within-guppy contrast addressed in previous publications. For this reason and because relative areas are highly variable, we did not account for the relative area abundance of each guppy color class. We simply addressed the question of contrast between each individual guppy patch and the adjacent visual background components.

### 2.4. Guppy Visual Contrast Calculations

We calculated guppy cone captures using the receptor noise model which estimates the difference in retinal stimulation ΔS between two stimuli [36]. We calculated both chromatic and luminance (achromatic) contrast, because color and luminance are processed separately in the retina and brain [37,38], and may be used for different purposes. For example, color may be used for mate quality assessment and luminance for species recognition. For general details and methods see [37]. We used the same guppy eye model parameters that we used previously [24,39]: the peak cone absorption wavelengths (λmax) for the four major single cone classes were UVS 359 nm; SWS 408 nm; MWS 465 nm; and LWS 560 nm. For the double cones (luminance), we used the sum of the absorption spectra for 543 nm and 560 nm cones. See [24] for details and justification for these and other parameters.

We calculated receptor noise contrast (ΔS) between every guppy color patch and either every substrate component (gravel grains, sand, silt, rocks, dead leaves) or every sidewelling spectrum (spacelight). There was very little courtship in early (*EL*) or small sunlit gap (*SG*) conditions (see results below), therefore our calculations were based on forest shade (*FS*) and cloudy (*OC*) conditions. For input, the radiances of each guppy spot radiance or each substrate component was calculated as the product of reflectance and either Forest Shade (*FS*) or Cloudy (*OC*) irradiance; spacelight spectra were already in radiance form. Given that guppies constantly change their orientation over the minute required for vertebrate chromatic adaptation (references in [40]), we used the mean of all spacelight spectra for the von Kries correction. These calculations did not account for the relative abundance of different substrate components or different guppy color patch classes because both substrates and guppy patch colors are highly variable in nature [21,22]. Our purpose is merely to examine the average contrasts over the various kinds of visual background components. We used the means of individual color patches rather than performing a full color pattern analysis [39,41,42] because we are simply asking if there are simple effects of orientation of the courting guppies on the average contrast between the visual background and guppy color patches. Ultimately one could do this for exact substrate proportions at a particular location and the male color patterns at the same locality. Chromatic ΔS was based upon the four guppy cone classes and achromatic ΔS was based upon the guppy double cones [24].

The analysis was based upon permutation tests and proceeded as follows: (1) We calculated ∆S between every guppy spot class and either every substrate or every spacelight spectrum, separately for *FS* (forest shade) and *OC* (cloudy). This yielded four distributions of ∆S representing the contrast between guppy spots and either substrates or spacelight. (2) We asked if guppies and either substrate or spacelight were significantly different by means of a permutation test in which we permuted group membership (guppy or visual background) and recorded the fraction of permuted values (*p*) when the observed mean ∆S equaled or exceeded the permuted mean ∆S. This is essentially asking by how much guppy males differ from either substrates or spacelight backgrounds, respectively. 

Next, we asked if the ∆S for guppy-spacelight (∆S_GS_) was larger than the ∆S for guppy–substrates (∆S_GB_, ‘B’ for background substrates), using another permutation test. (1) We calculated the absolute difference δ = | ∆S_GS_ − ∆S_GB_ | for all combinations of the two sets of ∆S; the mean of δ was taken as the observed value. (2) We permuted group membership (∆S_GS_ or ∆S_GB_) to obtain a distribution of permuted δ. (5) We compared the observed δ with the distribution of permuted δ to obtain the probability of obtaining the observed δ by chance. These are one-tailed tests because sexual selection should favor higher contrast. 

## 3. Results

### 3.1. Displays and Display Direction

We recorded behavioral sequence data from 82 males in Caigual and 128 males in Taylor. Means and SD of sequence lengths (numbers of direction codes in a male’s behavior sequence) were 3.03 ± 2.14 for Caigual and 4.09 ± 2.77 for Taylor. The data are skewed (Figure S1), as is typical for mating observations. We recorded only four sequences ending in copulation, two from each stream. We tested whether copulations were associated with longer display sequences by means of a one-tailed permutation test of differences in sigmoid display sequence lengths and found no evidence for display sequence length affecting mating success (*p* = 0.147, see Figure S2), although the sample size (4 out of 210) was very small.

**Figure 2:**
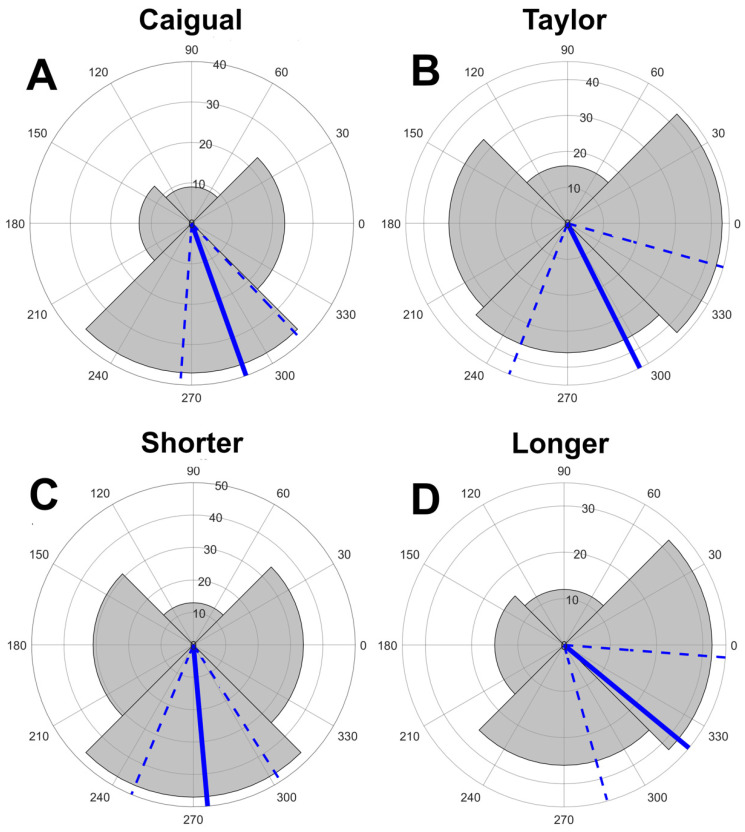
Distribution of direction records for the modal directions for each pair. Direction records for the two streams. Test results: (**A**): Caigual mn (mean vector) = 289.7°, s0 = 11.74, *p* = 0.0000, *n* = 82. (**B**) Taylor mn = 296.6°, s0 = 21.62, *p* = 0.0000, *n* = 128. Direction records for shorter (≤3) and longer (> 3) courtship sequences for both streams: (**C**): Shorter display sequences mn = 275.0°, s0 = 13.53, *p* = 0.0000, *n* = 125. (**D**) Longer sequences mn = 320.5°, s0 = 16.79, *p* = 0.0000, *n* = 85.

Courting pairs had a significant bias towards positions *D2* (0° towards far bank) and *D4* (270°, downstream) (Figure 2A). We cannot use conventional circular statistics to test for differences in mean direction here because the distribution is not continuous, having only four possible values; consequently, we used permutation tests to test for differences among groups and the Hodges-Adje test for nonuniform distributions (Figure 2A and Figure S3). The orientation of courting pairs used *D2* and *D4* more than expected, but Taylor pairs also used *D1* (towards near bank), Figure 2A). Overall (mean and 95% confidence direction) there was a strong preference for *D2* and *D4* (all *p* < 0.0001); pairs tended to orient where the female saw the male against the spacelight coming from the direction of the opposite bank or from downstream (Figure 1 and Figure 2). A permutation test on the difference in modal angles between the two streams found no significant difference, *p* = 0.77 (Figure S4). In summary, males tend to display to females such that they are seen against the spacelight away from the bank, instead of the sloping surface (usually gravel) of the near bank. When the female is facing away from the bank, she often faces more in the downstream than the opposite bank direction, (Figure 2A and Figure S3).

Longer displays may be associated with better males in the sense that they can display longer without the female losing interest and leaving him. Successful males may prefer being seen by females against the spacelight, thus longer displays should be oriented away from *D1* (near bank). We compared display directions between display sequences with 3 or fewer and 4 or more D codes. Pairs with longer displays tended to face relatively less downstream and more towards the opposite bank spacelight than those with shorter displays, hence away from the near bank (Figure 2B). A permutation test showed that the difference in orientation between long and short displays is significant, *p* = 0.024 (Figure S5). This is a one-tailed test because the overall mean is already towards spacelight, but we hypothesised that fewer pairs used *D1*, predicting a positive difference. Longer displays tend to be made more often towards the opposite bank than downstream (Figure 2B).

The sectors correspond to the direction zones in Figure 1C corresponding to each nominal direction (D) and the sector radii correspond to the number of fish with modal direction in that sector (note radial scale). *D1* is centered on 180° (female facing the near stream bank), *D2* centered at 0° (perpendicular to the near bank), *D3* centered at 90° (upstream), and *D4* centered at 270° (downstream). The blue lines indicate the circular mean and the dashed lines the 95% confidence limits. Titles include the circular mean and s0 (SE), the *p* resulting from a Hodges-Anje test (directional if *p* < 0.05) and the sample size. Females most often view a displaying male against spacelight from the opposite bank or downstream.

We compared the directions of the successful (copulation) versus the unsuccessful displays by means of a two-tailed permutation test. There was no evidence that orientation was different for successful displays, *p* = 0.30 (Figure S6), but there were only four males with displays ending in copulation.

### 3.2. Display Conditions

Most displays occurred in only one light environment, and only 2 out of the 210 switched between two environments. Courtship occurred predominantly in Forest Shade (*FS*) and Cloudy (*OC*) conditions, with little courtship occurring in the early morning (*EL*) or in sunlit patches (*SG*), see Table 1. For example, only 2/82 (2%) modal displays occurred in *SG* in Caigual and only 3/128 (2%) in Taylor. The commonly used light environments, *FS* and *OC*, were used differently between the two streams. Caigual males significantly preferred cloudy to shade conditions (1:1 expected, *p* < 0.0001) whereas Taylor males non-significantly preferred shade (*p* = 0.0603, Table 1). This may be related to the more open canopy in Taylor providing relatively more *OC* during our observations. A 2 × 2 Fisher-Freeman-Halton exact test with Forest Shade (*FS*) and Cloudy (*OC*) total counts as columns and streams as rows (Table 1) showed that the differential occurrence was significant, *p* < 0.0001. Pooling both streams, there was no preference between *FS* and *OC p* = 0.123, Table 1).

In order to investigate the possibility of particular direction sequences, we calculated the transition matrices between the four directions for both streams (see Table 2). We tested the transitions by χ^2^ with 1:1:1:1 expected at each row (Table S1). If a pair was at *D2* (facing towards the opposite bank spacelight), they most often changed (when they change) to *D3* (upstream) or *D4* (downstream); *p* = 0.068, 0.0148, 0.0454 for Caigual, Taylor, and both pooled, respectively (Table 3 and Table S1). If they were at *D3*, then they most often changed to *D2*; *p* = 0.0438, 0.0530, 0.0060. If they were at *D4*, then they most often changed to *D2*; *p* = 0.0028, 0.0004, < 0.0001. There was no obvious pattern when they were at *D1* (near bank); all *p* > 0.07 (Table S1). This does not show in Figure 2, because that table utilises the most common direction code for each male, and the transition matrices provide data on all directions utilised by all males.

### 3.3. Visual Contrast

In shallow water, where guppies display, we found the same downward irradiance spectra as described previously for terrestrial forested environments [31], Open/Cloudy (*OC*), Forest Shade (*FS*), Woodland Shade (*WS*), and Small Gaps (*SG*). The observed spectra did not include the Early (*EL*) spectra, probably because, by the time of the first observations at 7 am, the sun was higher than 10° above the horizon, too late in the morning for *EL* [31]. Sidewelling light spectra in shallow water next to the bank also showed the same four habitats (Figure S7). This is expected, because the mean overall gravel reflectance spectrum is fairly flat and so just reflects the ambient light spectrum, and light path lengths are too short for water color to affect the spectra. We rarely measured *WS* in these streams, mainly due to the high forest canopy cover and time of day (see [31]), and *OC* was mainly due to clouds rather than large (open) sunlit canopy gaps. 

The average spacelight (sidewelling) shows a significant drop in intensity at shorter wavelengths (Figure S8), and even more, as well as reduced variation, at 15 cm or deeper (Figure S9), especially compared to the close-to-bank spectra (Figure S7). This is expected from the attenuation (*K_d_*) spectrum, which shows increasing attenuation with decreasing wavelength (Figure S10). *WS* or *SG* results are not shown because the guppies in the study population courted mainly in *FS* and *OC* conditions (Table 1).

**Figure 3:**
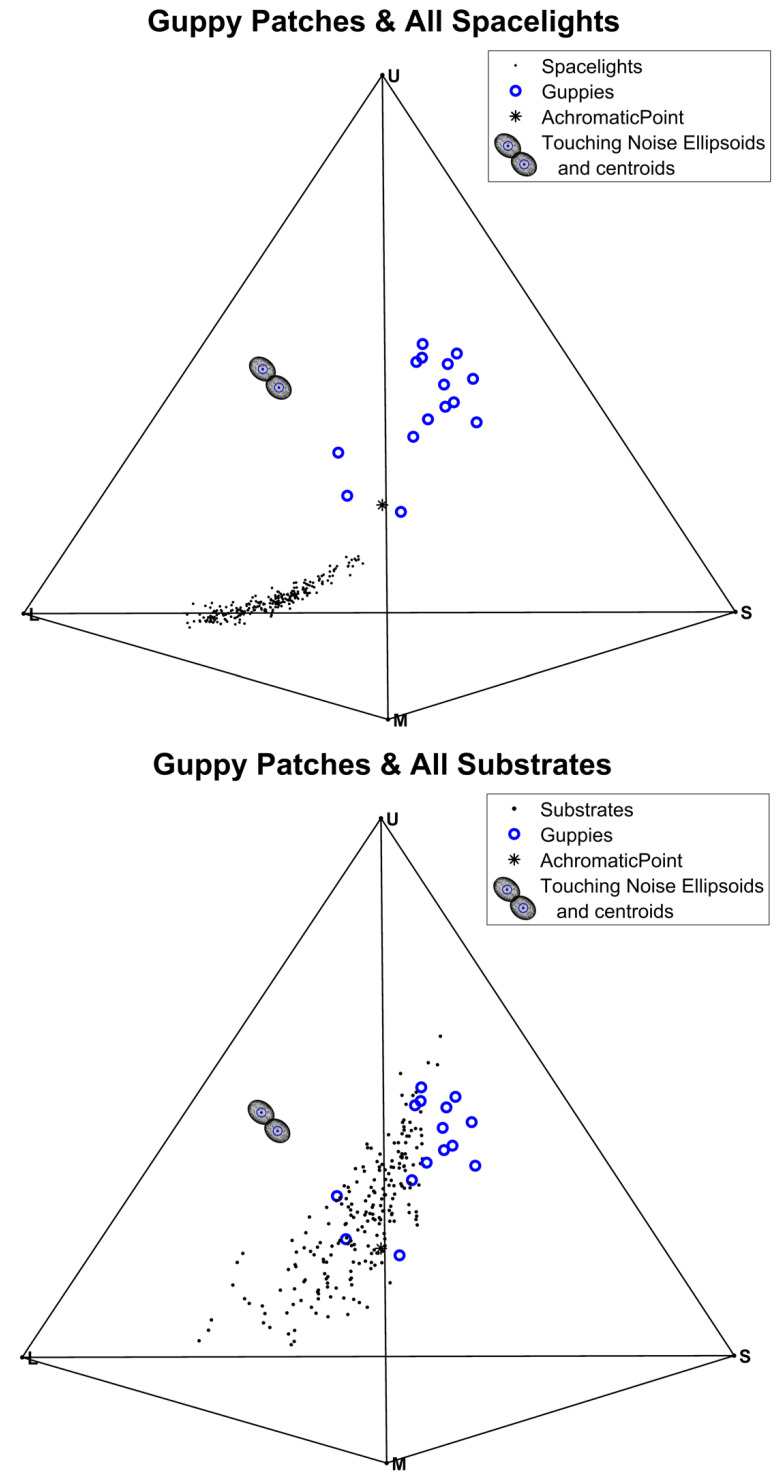
Chromatic (color) cone capture diagrams for guppy spots, spacelight and substrates in tetrahedral color space. Any point in the tetrahedron represents the relative values of four cone captures, which are the distances between a point and each tetrahedron face. Receptor noise causes randomly varying positions (ellipsoids) around a stimulus (ellipsoid centroid). The two ellipsoids in the figure were constructed using receptor noise values for each cone as 4D radii, then converted to the tetrahedron space. They are not spheres, because each cone has a different noise level. When two ellipsoids touch, their centroids are ∆S = 1 apart and the stimuli are just noticeably different (JND). The scale (distance between centroids) changes depending upon the relative position of any two stimuli; greater Euclidean distance when touching at the long axis and shorter when touching at a short axis. The two touching ellipsoids were placed in the tetrahedron so that they showed a larger JND than many parts of the tetrahedron and are there to give an idea of the limit of distances between distinguishable colours. Note the larger distance between the cloud of guppy points and spacelight compared to the distance to the substrates. A 3D movie is provided in the online (Video S1). Permutation tests show that guppy–spacelight and guppy–substrates are highly significantly different.

The simplest way to show the chromatic (color) differences between guppy patches, substrates, and spacelights is to plot their resulting guppy eye cone captures in a tetrahedron (Figure 3). Color depends upon the relative stimulation of four cone classes. The distance between any point in the tetrahedron and its four faces represents the relative value of the four channels; they sum to 1 when the tetrahedron height is 1. Consequently, every spectrum converted to four cone captures can be represented as a point in a tetrahedron [37], see Figure 3.

None of the guppy colors overlap with any of the spacelight spectra, but there is some overlap between guppy colors and some substrates (Figure 3). The spacelight yields very little stimulation of the UV (indicated by U) or SW (indicated by S) sensitive cones, hence those points are towards the bottom of the tetrahedron towards the LW (L) vertex and less close to the MW (M) cones. The shallower sidewelling spectra are influenced more by the substrates, which are close to being spectrally flat, so are closer to the achromatic point than the deeper samples. The substrates (gravel, sand, rocks, leaves, etc.) start just above the spacelight scans and rise up to the S and U vertices. This occurs because many substrate objects are spectrally flat and there are pebbles containing mica and other sources of structural colors which appear blue-gray.

The difference between the guppy colors and the spacelight is much greater than between guppies and the substrates (Figure 3). We tested this difference with the receptor noise model, rather than the Euclidean differences between the same spectra in the tetrahedron, because their geometry is different, although they can yield similar behavior predictions [43]. The scale difference results because receptor noise levels of each cone are different. As a result, the noise forms a 4D ellipsoid around the average stimulus (centroid) with 4 different radii (for each cone). This can be projected into a 3D Ellipsoid in the tetrahedron. Given that the noise range is not spherical, the relationship between Euclidean distance and ∆S differs in different directions in color space. When two appropriately sized noise ellipsoids touch (Figure 3), the distance between them is ∆S = 1, but the direction they touch affects the Euclidean distance between their centroids (see examples in [38]). This can be seen by the shape of the touching receptor noise ellipsoids in Figure 3; the U and S cones are nosier, therefore the ellipsoids are stretched along the U–S tetrahedron edge. The ellipsoids are provided in Figure 3 to give an idea of the scale of colour variation relative to ∆S = 1.

**Figure 4:**
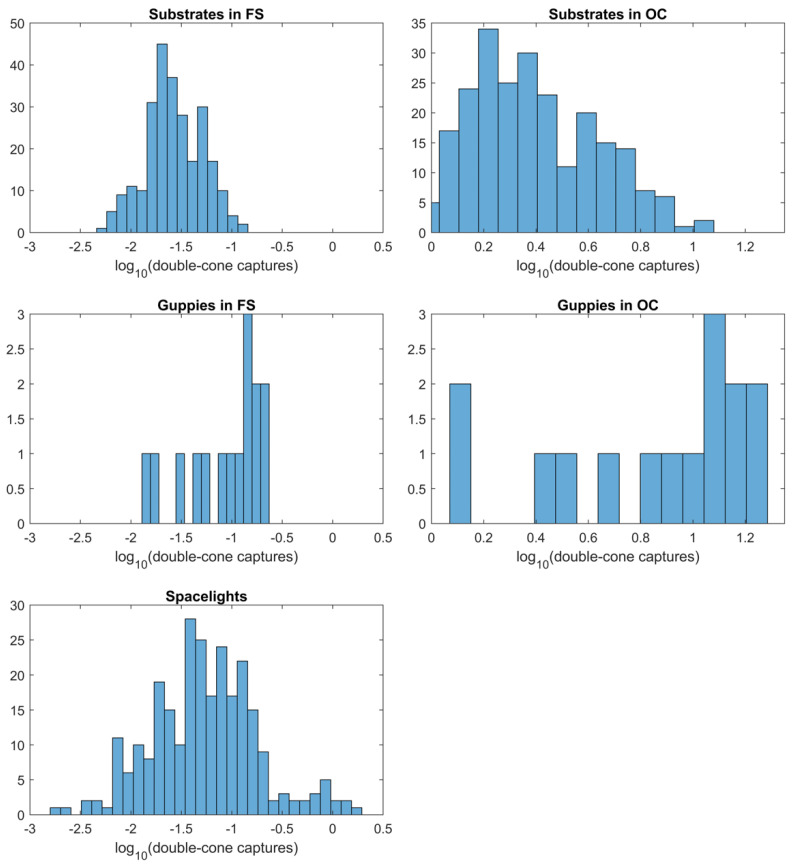
Distribution of luminance or achromatic (double cone) quantum captures for substrates, guppy patches and spacelight, after the von Kries correction. Note the very different horizontal scales for Forest Shade (*FS*) and Cloudy (*OC*) and between spacelight and *OC* data. This is because *FS* light is about 100 times less intense than *OC*.

Luminance (achromatic) from double-cone captures is shown in Figure 4. There is a large difference in the stimulation of the double cones between Forest Shade (*FS*) and Cloudy (*OC*) conditions, mainly because the total intensity of *OC* is about 100 times higher than *FS* [31]. The differences between guppy and backgrounds in achromatic stimulation are negligible in *FS* but strong in *OC* (Figure 4).

### 3.4. Tests of Contrast with the Visual Backgrounds (Substrates or Spacelights)

We calculated the chromatic and achromatic receptor noise contrast (ΔS) between guppy color and backgrounds: ΔS_GS_ for guppies against spacelights and ΔS_GB_ for guppies against substrate backgrounds (gravel grains, rocks, sand, silt, dead leaves), using either *FS* or *OC* light environments (see Figures S11 and S15). In order to test for chromatic and achromatic conspicuousness, we permuted group (guppy or background) membership 20,000 times and compared the distribution of permuted mean ΔS with the observed mean ΔS (Figures S12 and S16). Chromatic tests were one-tailed, because we expect the uniform spacelight to provide much less similarity to the guppies than the substrate purely on the basis of optics. Achromatic tests were two-tailed, because both guppies and parts of the substrate have high reflectance, hence no mechanistic a priori expectation. 

The chromatic differences between guppies and backgrounds (ΔS) are all highly significant (*p* < 0.00001) for both light environments (*FS* and *OC*, Table 3 and Figure S12), with ΔS >>1 (Table 3 and Figure S11), indicating strong chromatic contrast between guppies and both spacelight and substrates. This is consistent with the relative distances between the guppy, spacelight, and substrate clouds of tetrahedral points (Figure 3). This is expected from the results of sexual selection; male guppies should evolve more contrasting coloration no matter what visual background they are seen against [21].

However, the achromatic contrast varied with light environment (Table 3 and Figure 4 and Figure S16). In *OC* the contrast was very large (ΔS >> 1) and highly significant while being large but not significant in *FS*. The *FS* result follows from *FS* being 100× less intense than *OC* light [31], resulting in the range of double cone stimulation by the various spacelights completely overlapping that of double cone stimulation by guppy spots in *FS* (Figure 4). The mean ΔS in *FS* is still high (Table 3) because the achromatic differences between guppies and spacelights are high but similar to the within-spacelight (permuted) ΔS variation. This occurs because the double-cone captures have similar ranges (Figure 4). This did not occur in *OC* light. This is why the permutation test for guppy–spacelight ΔS in *FS* is not significant while that for *OC* is significant (Table 3 and Figure S16).

Of greater importance relative to orientation behavior is whether or not the contrast is greater against spacelight than substrates, given the behavioral observations. We tested whether the guppy–spacelight ΔS_GS_ were greater than the guppy–substrate ΔS_GB_ by calculating the differences δ = ΔS_GS_ − ΔS_GB_ for both chromatic and achromatic δ (Figures S13 and S17). When δ > 0, the contrast against spacelight is greater than against substrate backgrounds. To test the contrast difference, we compared the observed mean δ with the distribution of mean δ resulting from 20,000 permutations of group membership (ΔS_GS_ or ΔS_GB_, Figures S14 and S18). Once again, the results were highly significant (Table 3 and Figures S14 and S18) except for achromatic δ in *FS*. For chromatic δ under both light environments and achromatic δ under *OC*, δ >> 1, but for achromatic δ under *FS*, δ = −1.04, slightly negative. Although the achromatic *FS* δ is significant, indicating that guppy luminance in *FS* is less than spacelight, its magnitude is close to JND (ΔS = 1). This suggests that the slightly lower achromatic conspicuousness under *FS* might not be noticeable or important to guppies. In summary, as suggested by Figure 3, the chromatic contrast (ΔS) between guppies and spacelight (ΔS_GS_) is about twice as large as between guppies and substrates (ΔS_GB_) (Table 3). The achromatic contrast differences are similar in Forest Shade (*FS*), but ΔS_GS_ is three times larger than ΔS_GB_ under cloudy conditions (*OC*). There is a chromatic advantage to being seen against spacelight rather than substrates (δ), but only an achromatic advantage under cloudy conditions (Table 3). It is clear that the orientation of the courting pair as well as the light environment makes a difference in visual contrast between the male and the visual background, as seen by the female.

## 4. Discussion

Males display relatively more often under cloudy conditions (*OC*) in Caigual and relatively more often in Forest Shade (*FS*) in Taylor, although guppies in both populations prefer cloudy conditions over *SG* and *EL* (Table 1). This might be related to predator risk from fish, snakes, and fish-eating birds. *FS* is darker than *OC* and *SG*, making guppies relatively more difficult to see in *FS*. In addition, the greenish light of *FS* results in less total guppy reflectance (brightness) for a given total irradiance compared to *OC*, reducing visibility further. The relative difference in light usage is probably not due to availability, because Taylor has a relatively more open canopy than Caigual due to experimental canopy thinning [29]. Relative availability depends upon the size of canopy gaps [31]. Consequently, Taylor has relatively less stream area in *FS*, which predicts relatively more passive use of *OC* in Taylor, the opposite of what we observed (Table 1). Moreover, the *OC* light environment may be perceived as being associated with higher predation risk [30], hence guppies may be avoiding it in space and time during courtship when attention to potential predators is reduced. In any case, under both light environments, guppies contrast significantly against both substrates (Table 3 and Figures S7, S11, S12 and S16) and spacelight, both chromatically and achromatically, with greater contrast against spacelight than substrates (Table 3 and Figures S13, S14, S17 and S18).

**Figure 5:**
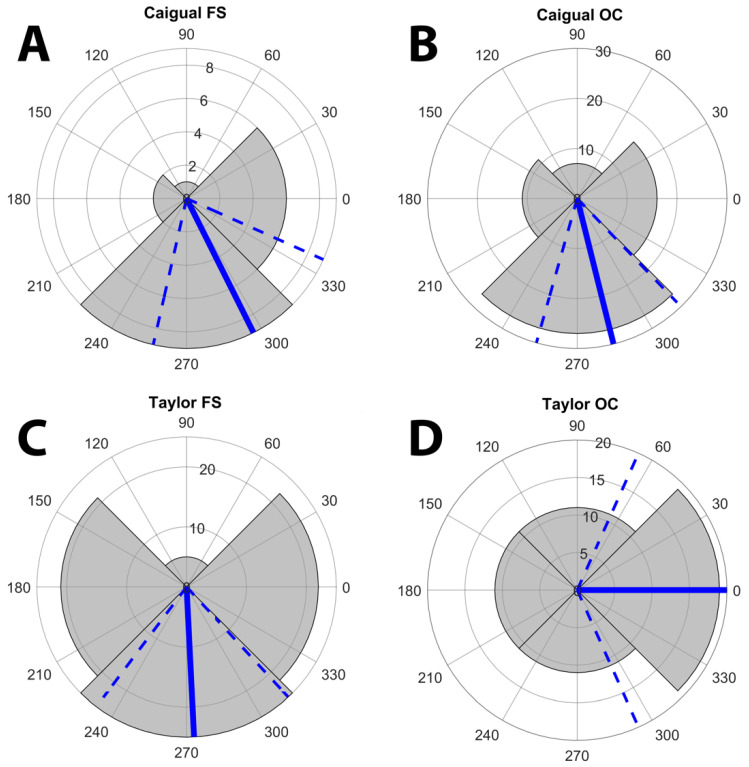
Direction data separated by both stream and light environment: *FS* (forest shade) and *OC* (cloudy). Symbolism as in Figure 2. The was no significant difference between *FS* and *OC* in Caigual, but there was a significant difference in Taylor. Test results: (**A**) Caigual *FS* mn = 296.6°, s0 = 18.57, *p* = 0.022, *n* = 18. (**B**) Caigual *OC* mn = 284.0°, s0 = 14.55, *p* = 0.0000, *n* = 61. (**C**) Taylor *FS* mn = 272.9°, s0 = 18.76, *p* = 0.0000, *n* = 73. (**D**) Taylor *OC* mn = 0.0°, s0 = 33.59, *p* = 0.0012, *n* = 52.

Given the differences between uses of ambient light (Table 1) and differences between chromatic and achromatic contrast (Figure 3 and Figure 4 and Table 3), we examined the distribution of directions separately for light environments and streams. All four combinations showed a preference for the female seeing the male away from the near stream bank, but the pattern is less consistent for Taylor than it is at Caigual (Figure 5). There is no difference in modal orientation for Caigual (*p* = 0.67) but a significantly greater tendency for direction *D2* (opposite bank spacelight) in *OC* (Cloudy) in Taylor (*p* = 0.032, Figure S19). There is always a chromatic advantage for a male being seen against spacelight relative to substrates, but, although this is true for achromatic contrast in *OC*, there is no achromatic advantage in forest shade. This shows the importance of the ambient light in courtship, even when the pair is choosing the visual background. It also suggests that both chromatic and achromatic contrasts are important in guppy courtship. However, these data do not address the question of whether different forms of contrast have different functions, for example, chromatic for mate assessment and achromatic for mate detection and tracking. 

Courting guppy pairs most often orient so that the female sees the male against the spacelight coming from the direction of the opposite bank or from downstream, rather than towards the near bank (Figure 2); she views him against spacelight rather than stream bank substrates. The substrate is more visually complex than the spacelight (Figure 1 and Figure 3). Spacelight and substrate visual backgrounds differ greatly in the spatial scale of color and luminance variation; little spatial variation in the spacelight and high variation in the substrates due to distinct multicolored objects [21]. There are at least six possible and non-exclusive reasons for our observed courting pair orientation: visual contrast, predator vigilance, predator risk, female pheromones, male vibrations, and energetics.

First, males may be maximising their visual contrast because chromatic contrasts are greater against spacelight than substrates under both *FS* and *OC* light conditions (Table 3 and Figure 3, Figure S11) whilst achromatic contrasts are only greater in *OC* conditions (Table 3 and Figure 4, Figure S15). The relative advantage of orientation against spacelight, measured by δ, is significantly positive for all chromatic δ and light conditions (Table 3 and Figures S13 and S14) and significantly positive for achromatic δ under *OC* (Table 3 and Figures S17 and S18). Under *FS*, achromatic δ is significantly negative but the magnitude is −1.04, which is almost the same as a JND. This suggests that although there is a small net achromatic disadvantage in *FS*, it may not be noticeable by a female. In addition, chromatic and achromatic contrast is enhanced when guppies move, because, against spacelight, the color pattern of the background adjacent to the guppy remains roughly constant but changes with every movement against the substrate, as different substrate components become adjacent to the guppy edge. Moreover, detection of objects is more difficult on more complex backgrounds [44,45,46,47], and discrimination is more difficult between complex textured objects than between plain ones [48]. For all these reasons males may be more visible and easier to track against spacelight than against the textured substrate of gravel, dead leaves and rocks.

Second, orientation may affect the efficacy of predator vigilance, because predators could come from offshore. If the male is between the female and the spacelight (locations *D2–D4*, Figure 1) then he has an unblocked view of possible approaching predators and rivals, whereas half of his visual field would be obstructed if he were next to the bank. Females are larger, so they also will have a good view of predators if she is closer to the bank than the courting male.

Third, orientation can affect relative predator risk especially for females. An attack from the depths is more likely to involve the closer and more conspicuous male (*D2–D4*) than the female if the male is between her and the open water. However, if the male is between the bank and the female (*D1*), then the female would be the first to be attacked and would also be distracted by the courting male in the opposite (bank) direction. Thus, this orientation may shift the risk towards males. In addition, male color patterns evolve to be similar in patch size to visual backgrounds (Endler 1980), and they are less contrasting against substrates (δ, Table 3), so male predation risk is reduced if they are seen against gravel rather than the spacelight (*D2–D4*). 

Fourth, orientation affects female pheromone transmission. Sexually receptive females release pheromones which are attractive to males. Non-receptive females release few or no pheromones [49]. As a result, males are attracted to receptive females but may ignore non-receptive females not releasing pheromones [50]. Females looking towards males downstream (*D4*) or more downstream than across (*D2*) may maximise the female pheromones flowing towards the male, making courtship more efficient or rapid, and hence possibly minimising predation risk during courtship.

Fifth, orientation affects male vibratory and pheromone signal efficacy. The sigmoid display involves oscillations around the body long axis and jumps towards and away from the female; she receives those signals in her lateral line. While females facing males downstream (*D4*) maximize the detection of female pheromones by males, the same orientation decreases the detection of male pheromones and male vibrational signals by females, because the vibrations and male pheromones need to travel against the current. Non-visual male-to-female signals would work better if the female viewed the male across the stream (*D2*), allowing some male pheromone and lateral line signals to reach the female. The relative position of the male and female during courtship is complex (Baerends et al., 1955). This, in addition to both orientation and water flow, modifies the relative efficiency of different sensory modes at different directions. Each mode has a different optimal orientation and can be used at different stages of the display. This suggests that the orientation of the pair should change with different mode usage during courtship in order to communicate through as many channels as possible. For example, males might try to entice females close enough to start the display by releasing pheromones upstream of her, then shift direction. If so, *D2* and possibly *D3* might precede *D4*. Unfortunately, we could not reliably score all the courtship stages except the sigmoid display because guppies are small and we were watching them from the stream bank. Hence, our observations only apply to sigmoid displays. The observed transition matrices (Table 3 and Table S1) show that if the pair is in *D3–D4*, it stays in these orientations. If the pair is in *D1* (female facing the bank), then they change to *D3–D4* at random. Considering the relative efficiency of visual vs. non-visual male signaling, it is suggestive that pairs in *D4* (female looking downstream) often change to *D2*, which would make non-visual male signaling more practical once the visual signaling resulted in a favorable reaction. Sequence form, sensory channel modes, and direction changes may both be important during courtship at different stages.

Sixth, orientation may affect the energetics of courtship. Positions *D1* and *D2* are probably the least energetically costly, because the water flow is along the body long axis (Figure 1B), so less energy is required to maintain position than if the body axis is facing perpendicular to the flow (*D3, D4*). *D2* may be better than *D1*, because in *D2* the female is closer to the bank and therefore closer to the boundary layer and experiencing relatively less flow. However, the distance between the pair may be too close for this to make a significant difference. Moreover, guppies tend to stay in low flow areas in small embayments, so energetic costs may not make as much of a difference as if they were in the main stream flow. The relative importance of these six factors in courtship orientation would repay further investigation.

Our general conclusion is that the orientation of courting guppy pairs is not random but is related to the visual background and the light environment. The direction of orientation is usually such that the female sees the displaying male performing a sigmoid display against spacelight rather than the much more visually complex substrate. This makes him relatively more conspicuous to females with respect to color (larger chromatic ∆S) but only with respect to luminance (achromatic ∆S) under *OC* light, which is brighter than *FS*. The same orientation may reduce the visibility of males to predators attacking towards the bank, because predators are more likely to see the male against the gravel of the near bank with both lower contrast (Figure 3) and similar color pattern patch size [21]. Orientation affects other sensory inputs (pheromones, vibratory signals) as well as courtship energetics. All of these factors affect the direction of evolution of courtship orientation and courtship sequences. Much needs to be done to explore the relationship between mating orientation relative to the sensory environment. This conclusion could apply to any organism which courts against a visually variable background and any sensory system with directional transmission.

## Figures and Tables

**Table 1:** Counts of light environments used during display sequences.

Light Environment	SG * Small Gaps	*EL* Early	*FS* Forest Shade	*OC* Cloudy	Total	χ^2^ 1:1	*p*
**Caigual**	2	1	18	61	**82**	23.41	0
**Taylor**	3	0	73	52	**128**	3.53	0.06
**Total**	**5**	**1**	**91**	**113**	**210**	2.37	0.123

Light (*FS-OC*) by Locality 2 × 2 χ^2^ = 24.85 with 1 df, *p* = 0.000001. Light (all) by Locality 2 × 4 Fisher-Freeman-Halton exact test χ^2^_(3)_ = 27.14, *p* < 0.000001. * Light environment names as in [31].

**Table 2:** Transition matrices from direction sequences (*D1, D2, D3, D4* in Figure 1) and *p* for row χ^2^ with 1:1:1:1 expected.

	Caigual					Taylor					Both Pooled				
Direction	*D1*	*D2*	*D3*	*D4*	*p* for χ^2^	*D1*	*D2*	*D3*	*D4*	*p* for χ^2^	*D1*	*D2*	*D3*	*D4*	*p* for χ^2^
***D1***	5	13	8	15	0.1058	30	20	13	21	0.073	35	33	21	36	0.2008
***D2***	6	9	11	22	0.01	18	38	23	19	0.015	24	47	34	41	0.0454
***D3***	8	12	2	5	0.044	13	28	14	21	0.053	21	40	16	26	0.006
***D4***	14	20	2	17	0	21	33	9	36	0	35	53	11	53	<0.0001

**Table 3:** Permutation tests for ∆S between guppy patches and backgrounds (spacelight or substrates) and for differences δ between these two sets of ∆S.

	Forest Shade (*FS*)			Cloudy (*OC*)		
Test	Observed Mean ∆S or Difference δ	Permutation Test *p*	Supplemental Figure	Observed Mean ∆S or Difference δ	Permutation Test *p*	Supplemental Figure
Chromatic ∆S Guppy-Spacelight	13.2	<0.00001	Figure S11	12.9	<0.00001	Figure S11
Chromatic ∆S Guppy-Substrate	5.98	<0.00001	Figure S11	5.98	<0.00001	Figure S11
Chromatic δ	+7.25	<0.00001	Figure S14	+6.92	<0.00001	Figure S14
Achromatic ∆S Guppy-Spacelight	12.6	0.73	Figure S16	48.8	<0.00001	Figure S16
Achromatic ∆S Guppy Substrate	13.7	<0.00001	Figure S16	13.7	<0.00001	Figure S16
Achromatic δ	−1.04	<0.00001	Figure S18	+5.1	<0.00001	Figure S18

δ = ΔS_GS_ − ΔS_GB_, where ΔS_GS_ is between guppies and spacelights and ΔS_GB_ is between guppies and substrates. This measures the relative advantage of displaying against spacelight compared to substrates; positive favours spacelight, negative favours substrates. Although the achromatic δ in *FS* was negative and highly significant, its value is close to 1, the same as JND (ΔS = 1), suggesting little obvious advantage of using substrates in *FS*. All other δ >> 1.

## Data Availability

Data are part of the online material at https://www.mdpi.com/article/10.3390/vision6030056/s1.

## Supplementary Materials

The following supporting information can be downloaded at: https://www.mdpi.com/article/10.3390/vision6030056/s1, Figures S1–S19, Table S1, a movie of Figure 3 rotating for ease in viewing in 3D, and the data in a zip file. Video S1.

## References

[1] Brock C.D., Cummings M.E., Bolnick D.I. (2017). Phenotypic plasticity drives a depth gradient in male conspicuousness in threespine stickleback, *Gasterosteus aculeatus*. Evolution.

[2] Cole G.L., Endler J.A. (2015). Variable Environmental Effects on a Multicomponent Sexually Selected Trait. Am. Nat..

[3] Cole G.L., Endler J.A. (2016). Male courtship decisions are influenced by light environment and female receptivity. Proc. R. Soc. B Boil. Sci..

[4] Leal M., Fleishman L.J. (2004). Differences in visual signal design and detectability between alloatric populations of Anolis lizards. Am. Nat..

[5] Uetz G.W., Clark D.L., Roberts J.A., Rector M. (2011). Effect of visual background complexity and light level on the detection of visual signals of male Schizocosa ocreata wolf spiders by female conspecifics. Behav. Ecol. Sociobiol..

[6] Camacho C., Sanabria-Fernández A., Baños-Villalba A., Edelaar P. (2020). Experimental evidence that matching habitat choice drives local adaptation in a wild population. Proc. R. Soc. B Boil. Sci..

[7] Endler J.A., Théry M. (1996). Interacting effects of lek placement, display behavior, ambient light and color patterns in three neotropical forest-dwelling birds. Am. Nat..

[8] Heindl M., Winkler H. (2003). Vertical lek placement of forest-dwelling manakin species (Aves, Pipridae) is associated with vertical gradients of ambient light. Biol. J. Linn. Soc..

[9] Leal M., Fleishman L.J. (2001). Evidence for habitat partitioning based on adaptation to environmental light in a pair of sympatric lizard species. Proc. R. Soc. B Boil. Sci..

[10] Mameri D., van Kammen C., Groothuis T.G.G., Seehausen O., Maan M.E. (2019). Visual adaptation and microhabitat choice in Lake Victoria cichlid fish. R. Soc. Open Sci..

[11] Schultz T.D., Anderson C.N., Symes L.B. (2008). The conspicuousness of color cues in male pond damselflies depends on ambient light and visual system. Anim. Behav..

[12] Christy J.H., Baum J., Backwell P.R. (2003). Attractiveness of sand hoods built by courting male fiddler crabs, Uca musica: Test of a sensory trap hypothesis. Anim. Behav..

[13] Cummings M.E., Jordão J.M., Cronin T.W., Oliveira R.F. (2008). Visual ecology of the fiddler crab, Uca tangeri: Effects of sex, viewer and background on conspicuousness. Anim. Behav..

[14] Holmlund M., Östlund-Nilsson S. (2003). The artistic three-spined stickleback (Gasterosteous aculeatus). Behav. Ecol. Sociobiol..

[15] Uy J.A.C., Endler J. (2004). Modification of the visual background increases the conspicuousness of golden-collared manakin displays. Behav. Ecol..

[16] White T.E., Vogel-Ghibely N., Butterworth N.J. (2020). Flies Exploit Predictable Perspectives and Backgrounds to Enhance Iridescent Signal Salience and Mating Success. Am. Nat..

[17] Bolnick D.I., Shim K.C., Brock C.D. (2015). Female stickleback prefer shallow males: Sexual selection on nest microhabitat. Evolution.

[18] Brock C.D., Rennison D., Veen T., Bolnick D.I. (2018). Opsin expression predicts male nuptial color in threespine stickleback. Ecol. Evol..

[19] Houde A.E. (1997). Sex, Color and Mate Choice in Guppies.

[20] Waterbolk H., Brouwer R., Baerends G. (1955). Ethological Studies On Lebistes Reticulatus (Peters). Behaviour.

[21] Endler J.A. (1980). Natural selection on color patterns in Poecilia reticulata. Evolution.

[22] Endler J.A. (1978). A predator’s view of animal color patterns. Evol. Biol..

[23] Long K.D. (1993). Variation in Mating Behavior of the Guppy, Poecilia Reticulata, as a Function of Environmental Irradiance, Visual Acuity, and Perception of Male Color Patterns. Ph.D. Thesis.

[24] Kranz A.M., Cole G.L., Singh P., Endler J.A. (2018). Colour pattern component phenotypic divergence can be predicted by the light environment. J. Evol. Biol..

[25] Long K.D., Rosenqvist G. (1998). Changes in male guppy courting distance in response to a fluctuating light environment. Behav. Ecol. Sociobiol..

[26] Fernald R.D., Douglas R., Djamgoz M. (1990). The Optical System of Fishes. The Visual System of Fish.

[27] Douglas R.H., Hawryshyn C.W., Douglas R., Djamgoz M. (1990). Behavioral studies of fish vision: An analysis of visual capabilities. The Visual System of Fish.

[28] Lythgoe J. (1979). The Ecology of Vision.

[29] Kohler T.J., Heatherly T.N., El-Sabaawi R.W., Zandonà E., Marshall M.C., Flecker A.S., Pringle C.M., Reznick D.N., Thomas S.A. (2012). Flow, nutrients, and light availability influence Neotropical epilithon biomass and stoichiometry. Freshw. Sci..

[30] Endler J.A. (1987). Predation, light intensity, and courtship behavior in Poecilia reticulata. Anim. Behav..

[31] Endler J. (1993). The Color of Light in Forests and Its Implications. Ecol. Monogr..

[32] Mardia K.V., Jupp P.E. (2000). Directional Statistics.

[33] Mielke P.W., Berry K.J. (2001). Permutation Methods: A Distance Function Approach.

[34] Endler J.A. (1990). On the measurement and classification of color in studies of animal color patterns. Biol. J. Linn. Soc. Lond..

[35] Kirk J.T.O. (1994). Light and Photosynthesis in Aquatic Ecosystems.

[36] Vorobyev M., Osorio D. (1998). Receptor noise as a determinant of color thresholds. Proc. R. Soc. Lond. Ser. B Biol. Sci..

[37] Endler J.A., Mielke P.W. (2005). Comparing entire color patterns as birds see them. Biol. J. Linn. Soc. Lond..

[38] Kemp D.J., Herberstein M.E., Fleishman L.J., Endler J.A., Bennett A.T.D., Dyer A.G., Hart N.S., Marshall J., Whiting M.J. (2015). An Integrative Framework for the Appraisal of Coloration in Nature. Am. Nat..

[39] Sibeaux A., Cole G.L., Endler J.A. (2019). The relative importance of local and global visual contrast in mate choice. Anim. Behav..

[40] Endler J.A., Gaburro J., Kelley L. (2014). Visual effects in great bowerbird sexual displays and their implications for signal design. Proc. R. Soc. B Boil. Sci..

[41] Endler J.A., Cole G.L., Kranz A.M. (2018). Boundary strength analysis: Combining colour pattern geometry and coloured patch visual properties for use in predicting behaviour and fitness. Methods Ecol. Evol..

[42] van den Berg C.P., Troscianko J., Endler J.A., Marshall N.J., Cheney K.L. (2020). Quantitative color pattern analysis (QCPA): A comprehensive framework for the analysis of color patterns in nature. Methods Ecol. Evol..

[43] Fleishman L.J., Perez C.W., Yeo A.I., Cummings K.J., Dick S., Almonte E. (2016). Perceptual distance between colored stimuli in the lizard Anolis sagrei: Comparing visual system models to empirical results. Behav. Ecol. Sociobiol..

[44] Dimitrova M., Merilaita S. (2009). Prey concealment: Visual background complexity and prey contrast distribution. Behav. Ecol..

[45] Dimitrova M., Merilaita S. (2011). Prey pattern regularity and background complexity affect detectability of background-matching prey. Behav. Ecol..

[46] Hansen T., Giesel M., Gegenfurtner K.R. (2008). Chromatic discrimination of natural objects. J. Vis..

[47] Santiago C., Green N.F., Hamilton N., Endler J.A., Osorio D.C., Marshall N.J., Cheney K.L. (2020). Does conspicuousness scale linearly with colour distance? A test using reef fish. Proc. R. Soc. B Boil. Sci..

[48] Pas S.F.T., Koenderink J.J. (2004). Visual Discrimination of Spectral Distributions. Perception.

[49] Crow R.T., Liley N.R. (1979). A sexual pheromone in the guppy, Poecilia reticulata (Peters). Can. J. Zoo..

[50] Guevara-Fiore P., Stapley J., Watt P.J. (2010). Mating effort and female receptivity: How do male guppies decide when to invest in sex?. Behav. Ecol. Sociobiol..

