# Supplementary material for: Visual Background Choice and Light Environment Affect Male Guppy Visual Contrast
Source: Vision (Basel). 2022 Sep 7;6(3):56. doi: 10.3390/vision6030056 (PMC9500966; doi:10.3390/vision6030056)
Supplement: Supplementary file 1 [file vision-06-00056-s001.zip › Supplement/EndlerEtAlGuppyBackgroundChoiceSupplementFULL.pdf]

Supplemental material for:

Chosen visual background and light environment affects male guppy visual contrast

John A. Endler[1], Dara-Marie Raggay[2], Solomon Maerowitz-McMahan[3], David N. Reznick[3], and Rebecca C. Fuller[4]

[1]Centre for Integrative Ecology, School of life and Environmental Sciences, Deakin University, Waurn Ponds, VIC 3216, Australia

[2]Department of Life Sciences, University of the West Indies, St Augustine, Trinidad & Tobago, W. I.

[3]Department of Biology, University of California, Riverside, CA 92521 USA

[4]School of Integrative Biology, University of Illinois at Urbana-Champaign, Champaign, IL 61820 USA

address for correspondence: John.Endler@deakin.edu.au

There is also a movie showing the 3D version of figure 3.

Data are available in Dryad as separate files so that the data can be explored without the need for extraction from a PDF file.

SUPPLEMENTAL FIGURES AND TABLE

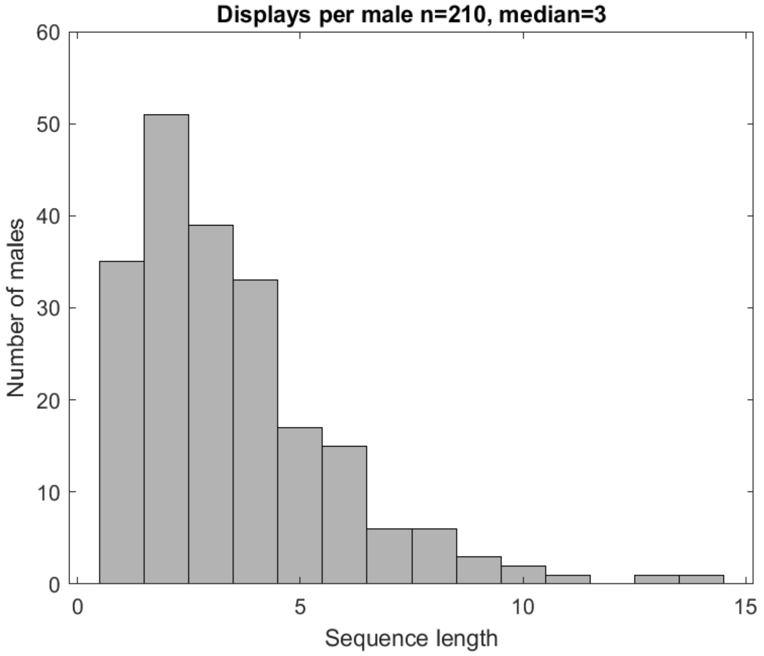

Figure S1. Distribution of the sequence lengths (number of displays D) among all males observed. Data from both streams.

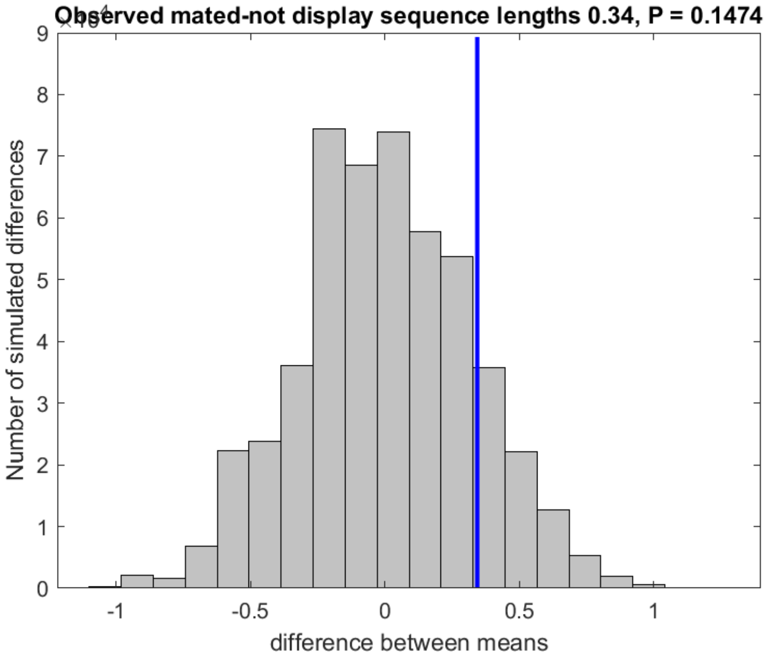

Figure S2. Permutation test of the mean difference in the number of displays (copulated-not). Each permutated mean was calculated over all difference combinations. Group membership (mated or not) was permuted by the MATLAB function randperm. The histogram shows the distribution of these differences in 200,000 permutations of group membership and the vertical bar is the observed mean difference. P comes from the relative numbers of simulated values at the observed value or higher (not the histogram bar areas). P=0.147, although there were only 4 displays in the mated group. This is a one-tailed test because we thought that mating success might be higher for longer display sequences.

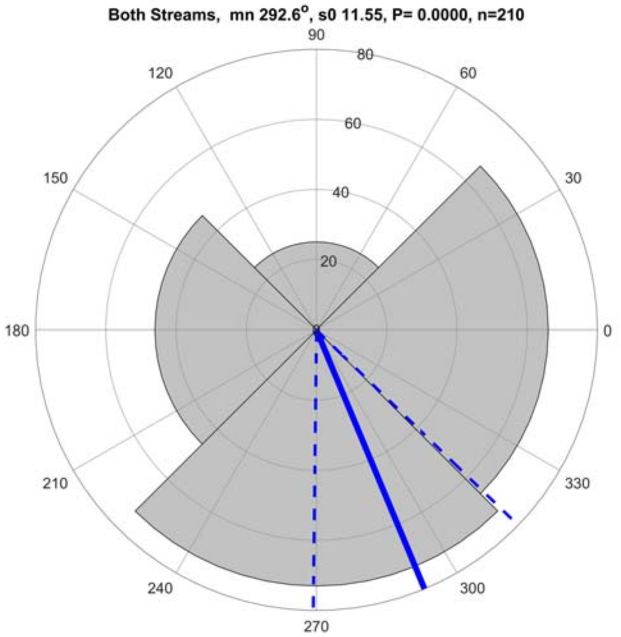

Figure S3. Distribution of modal courtship directions for the two streams pooled. As in Fig. 2, the gray sectors are the distribution of directions D1 to D4. D1=180°, D2=0°, D3=90°, D4=270°and the blue line indicates the circular mean direction and the dashed line the 95% circular confidence limits. Title includes the circular mean and s0 of the modal angles and the result of a Hodges-Ajne test for non-uniformity (a measure of directionality).

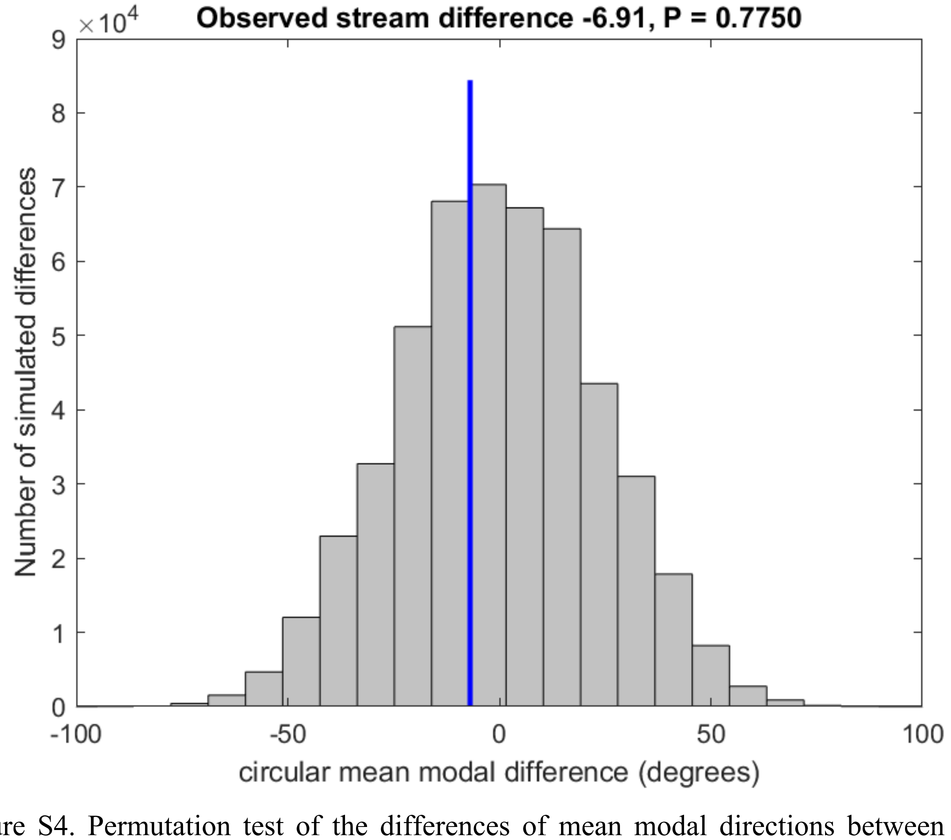

Figure S4. Permutation test of the differences of mean modal directions between Caigual and Taylor. Same symbolism as in Fig. S2 except + is counterclockwise (higher angle). No significant difference (2-tailed), P=0.77.

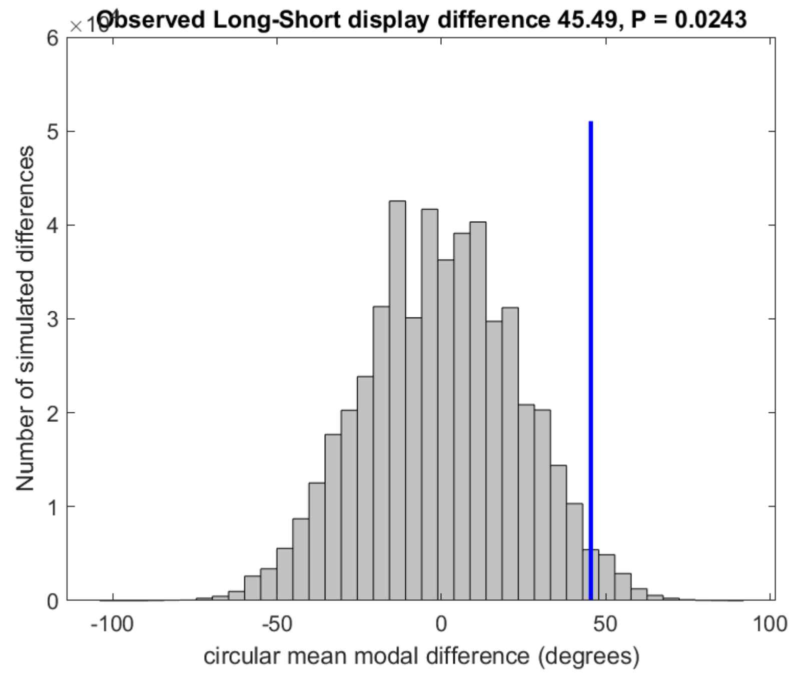

Figure S5. Permutation test of the difference in modal directions between longer (5 or more) and shorter (4 or less) display lengths. Longer displays are significantly relatively closer to position 2 (larger angles), P=0.024 (one-tailed).

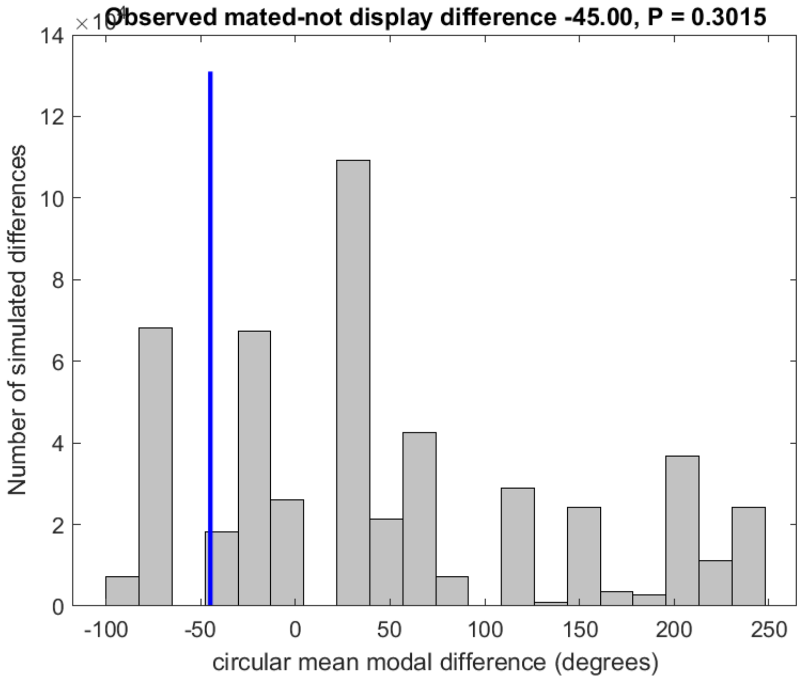

Figure S6. Permutation test between modal angles of sequences ending in copulations and those not ending in copulation. There were only 4 males obtaining copulations, which is why the permuted differences are so discontinuous. No significant difference, P=0.30. Two-tailed test owing to very unequal sample sizes.

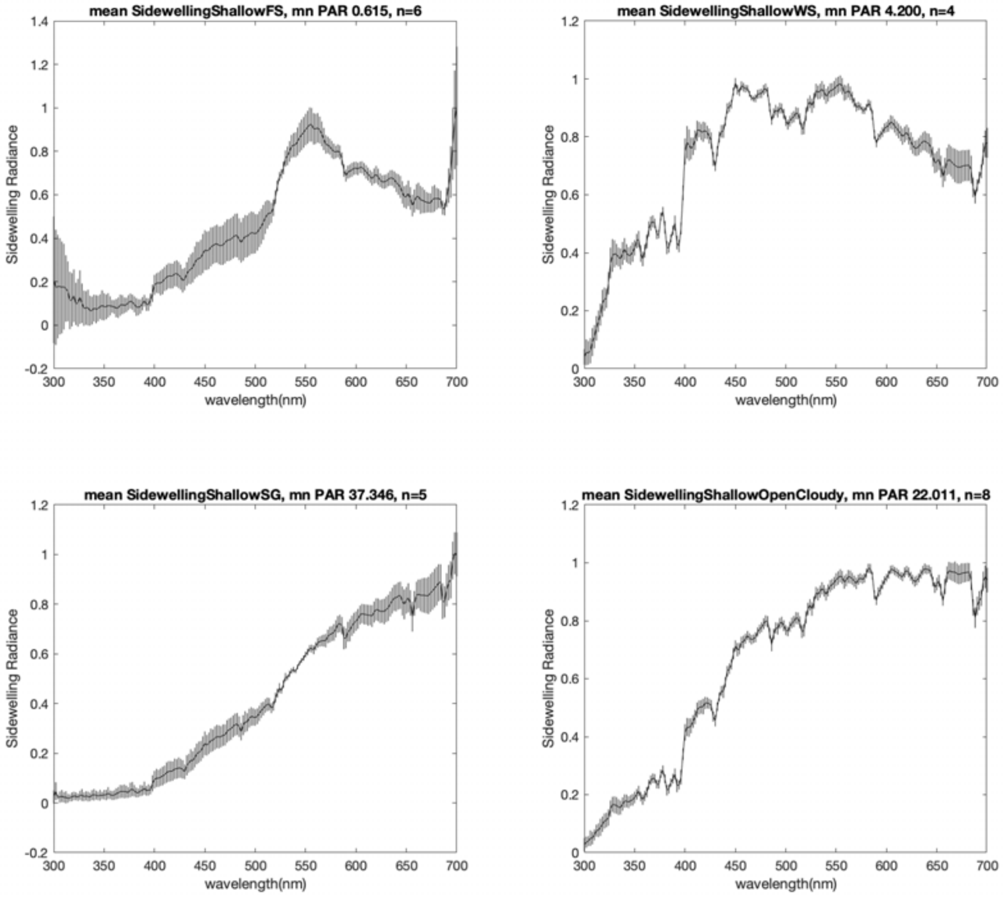

Figure S7. Mean and SD sidewelling radiance measured towards the stream bank in shallow water, separated by light environments (FS=Forest Shade, WS=Woodland Shade, SG=Small Sun Gaps, OC=Cloudy or Open; Measurements were taken at the same depth as courting guppy pairs.

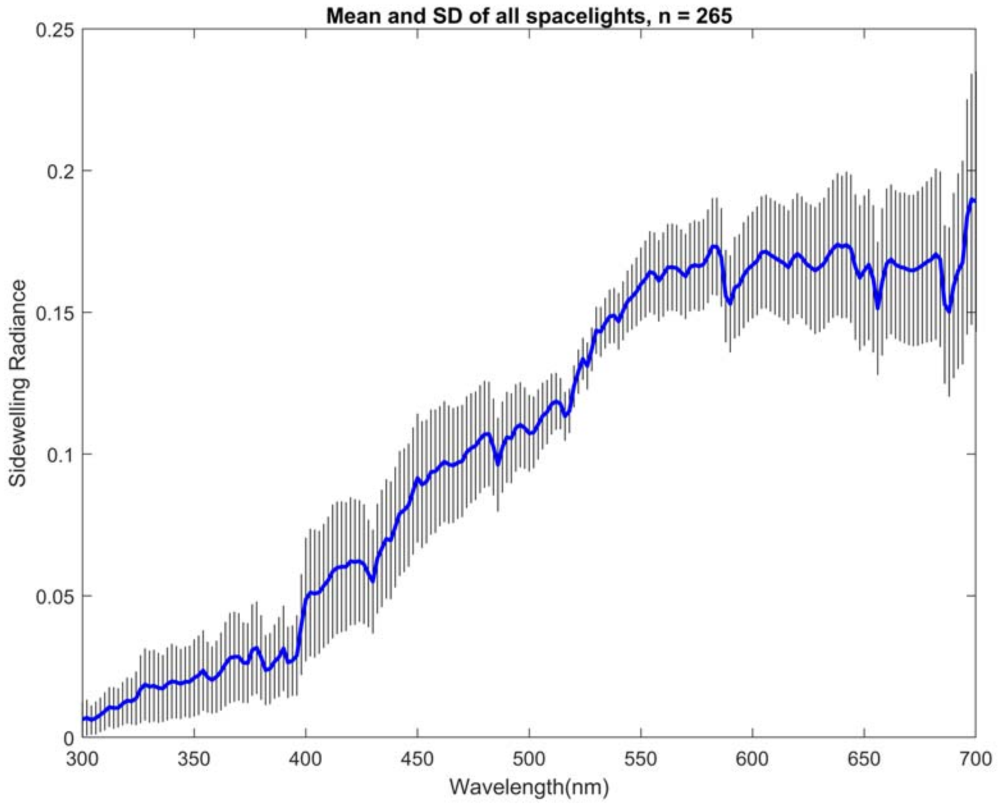

Figure S8. Mean and SD of all spacelight (sidewelling light) scans. The blue line is the mean and the vertical lines are the mean ± 1 SD. The noise results from a combination of the geometry of Snell's window with depth and the nearness of substrates.

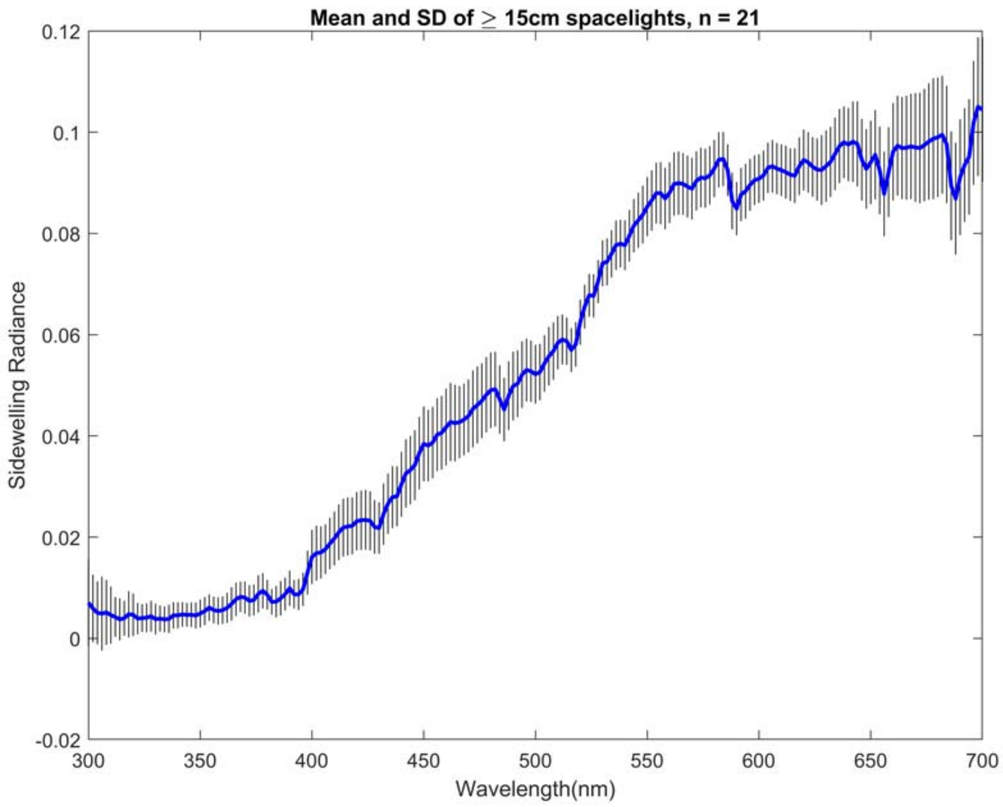

Figure S9. Mean and SD of spacelight measured at 15cm or deeper to minimise the effects of Snell's window (ambient light above the water) and any nearby banks. Same convention as Fig. S8. Note different vertical scale from Fig S8 (deeper is darker).

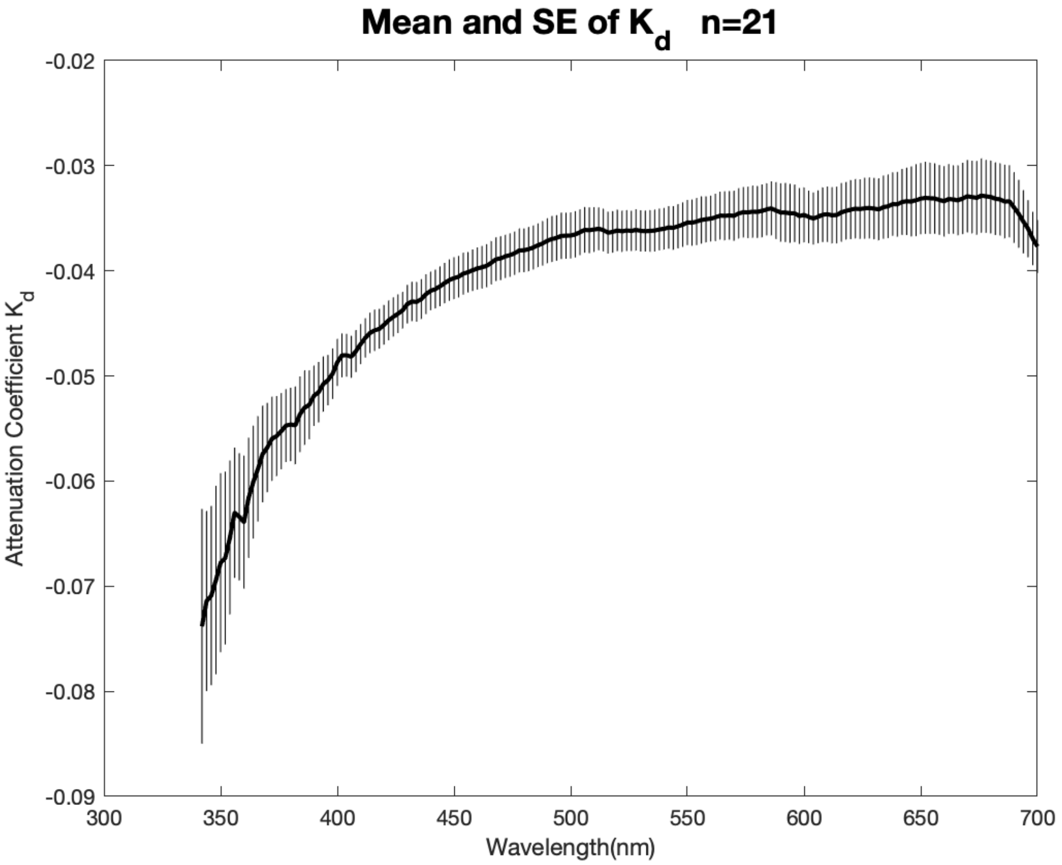

Figure S10. Mean and SE of the downward attenuation K_d at each wavelength. This was calculated from 21 K_d spectra at multiple points among both streams. Values below 350nm were too noisy to use and hence are not shown. Lower values mean more attenuation.

Chromatic ΔS

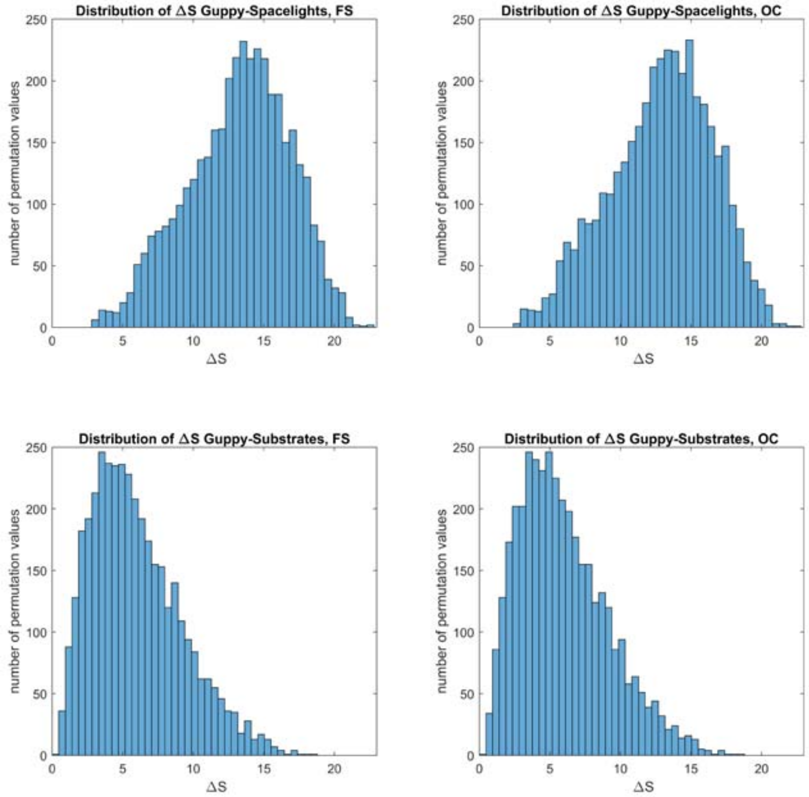

Figure S11. Distribution of observed chromatic ΔS between all guppy patches and all spacelights (first row) or all substrates (second row), for FS (Forest Shade) or OC (Cloudy) conditions (columns).

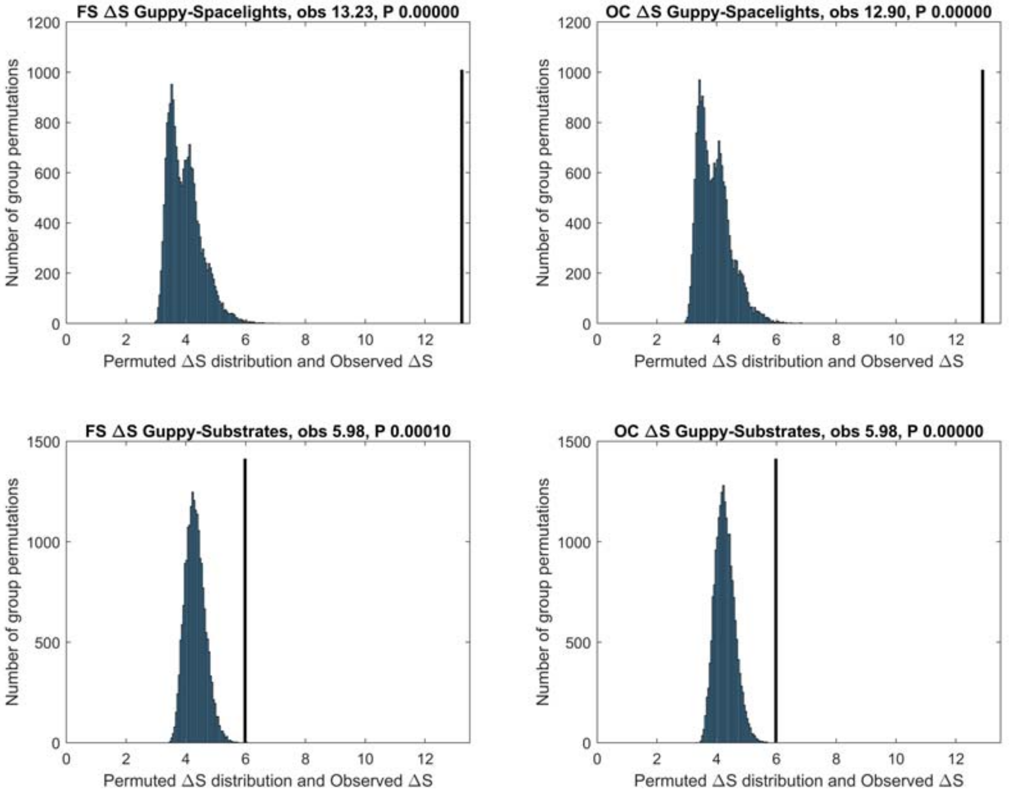

Figure S12. Permutation tests comparing Guppy spots and either spacelight or substrates, separately under FS (Forest Shade) or OC (Cloudy). The vertical black line is the observed mean chromatic ΔS between guppy and background (see also Fig. S11), and the histogram shows the distribution of the mean ΔS for each of 20000 permuted memberships between guppy and spacelight or substrates.

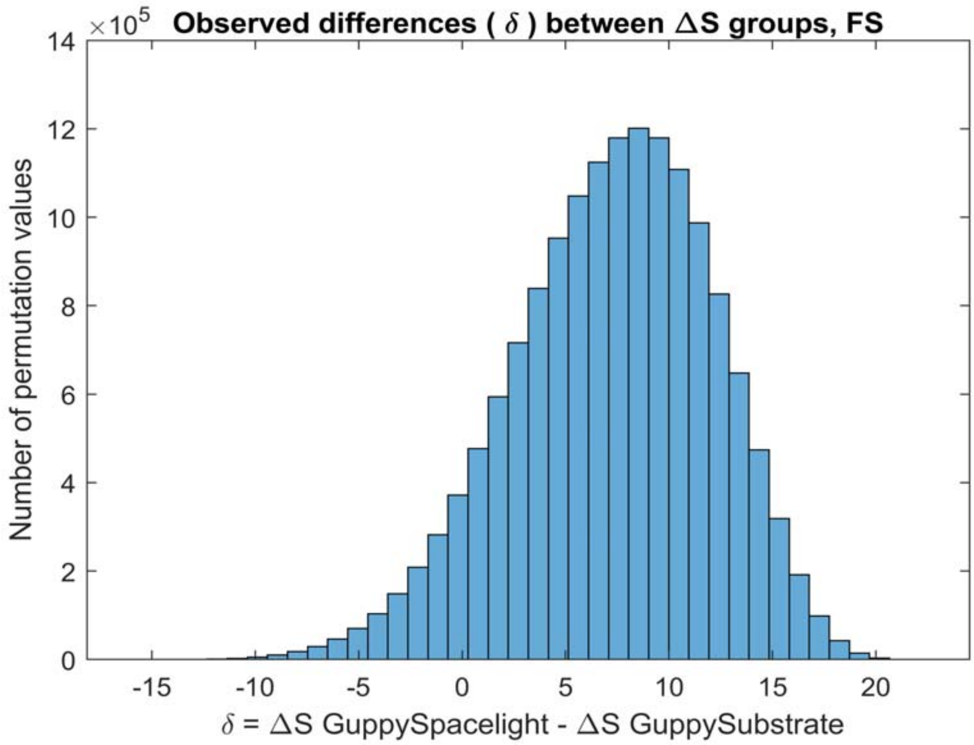

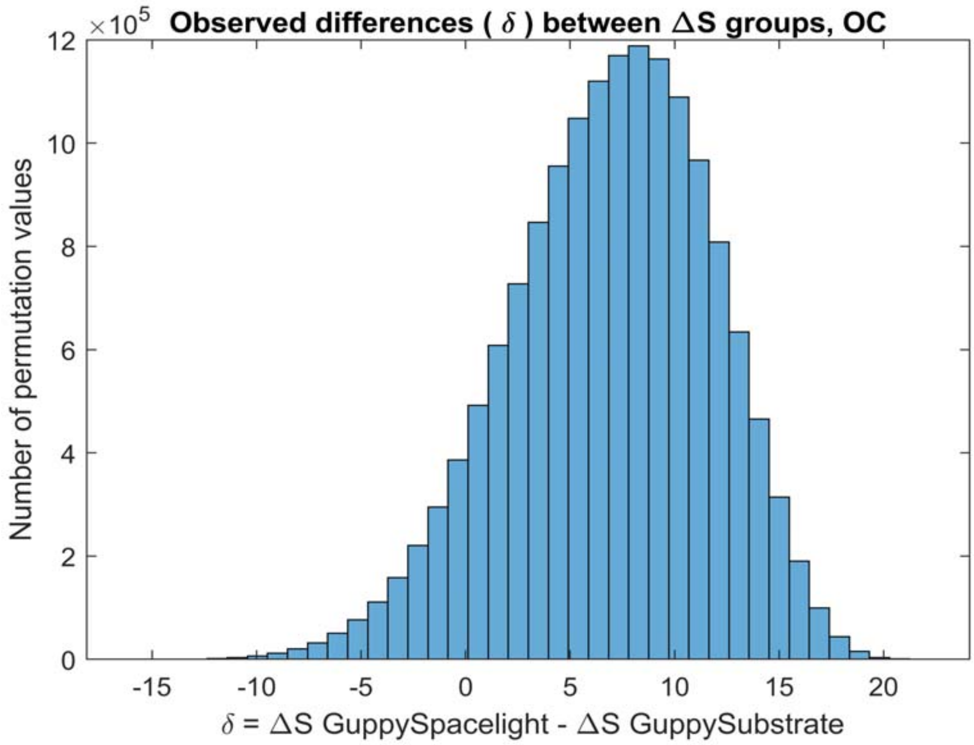

Figure S13. Distribution of the chromatic ΔS differences $\delta = \Delta S_{GS} - \Delta S_{GB}$, where ΔS_{GS} is measured between guppy and spacelight and ΔS_{GB} is measured between guppies and substrate backgrounds, in FS and OC light environments. A positive value indicates that guppy spots are more conspicuous against spacelights than substrates and *vice-versa*.

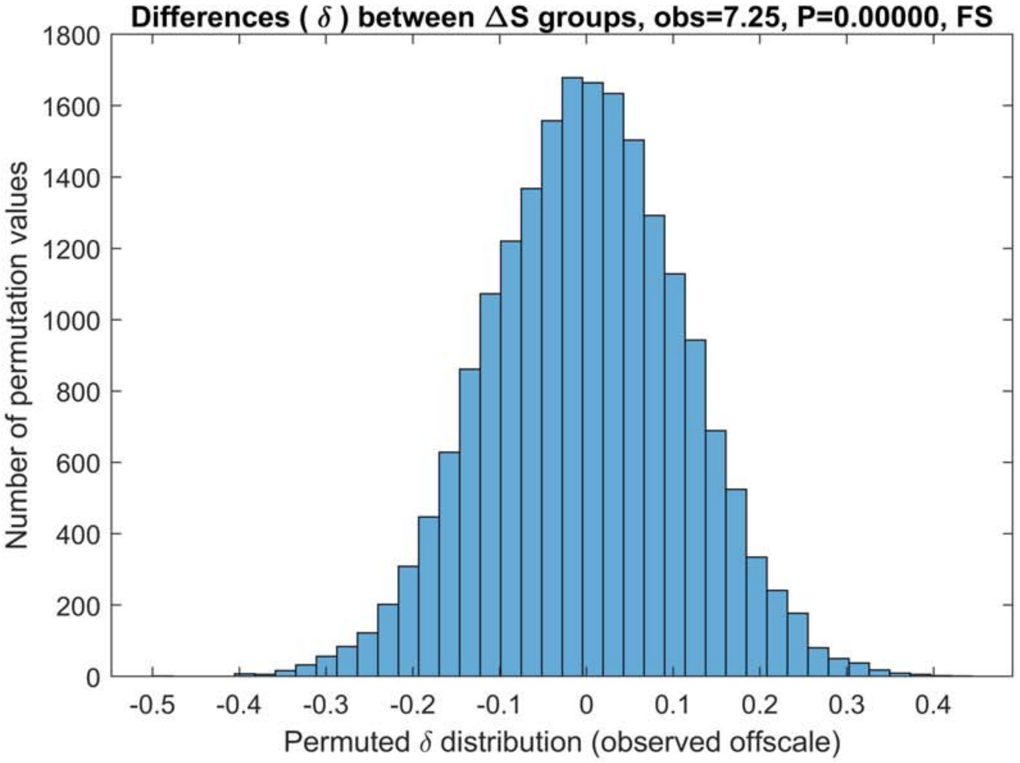

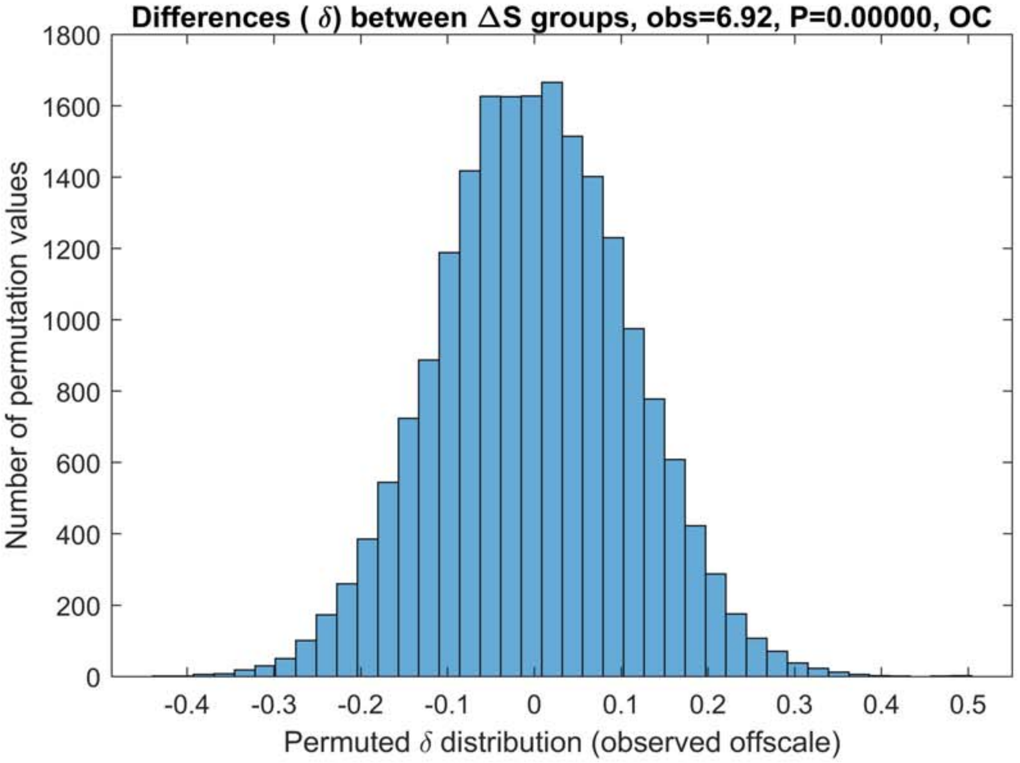

Figure S14. Results of the permutation tests of the differences (δ) in chromatic ΔS measured by $\delta = \Delta S_{GS} - \Delta S_{GB}$, where ΔS_{GS} is measured between guppy and spacelight and ΔS_{GB} is measured between guppies and substrate backgrounds. Histograms show the permutated differences. ***The observed mean absolute differences are not shown here because they are past the right margin;*** values are 7.25 and 6.92 for FS and OC, respectively.

Achromatic ΔS

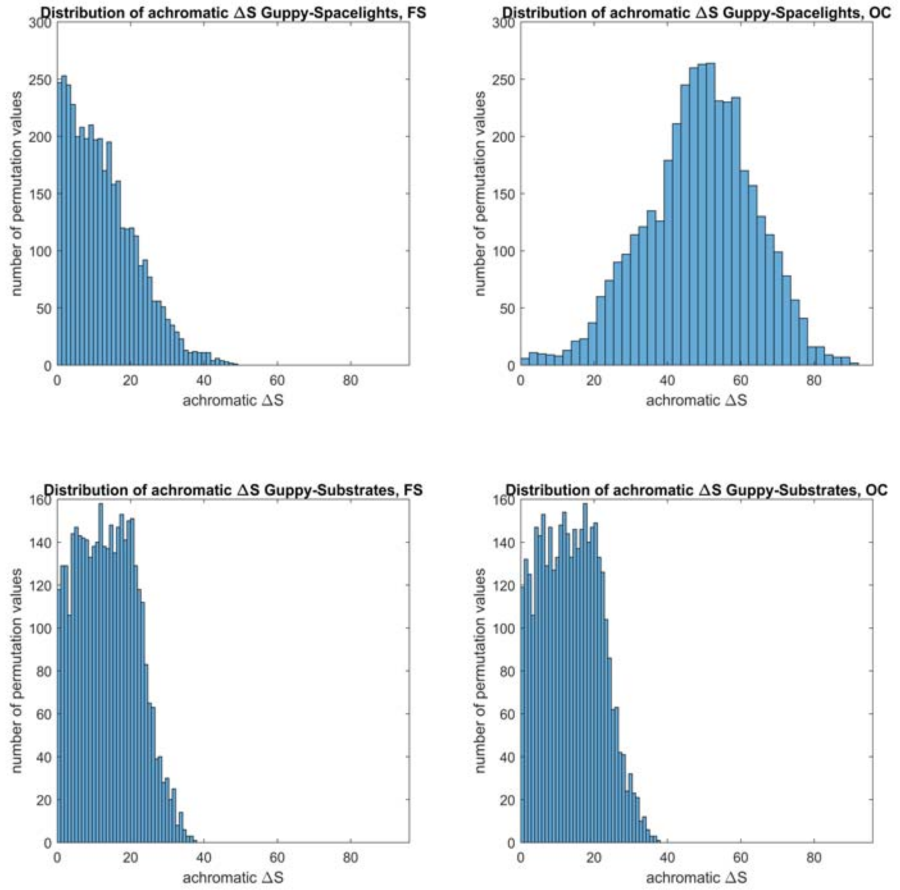

Figure S15. Luminance (Achromatic) ΔS between guppies and spacelight and between guppies and substrates. Same symbolism as in Fig. S11.

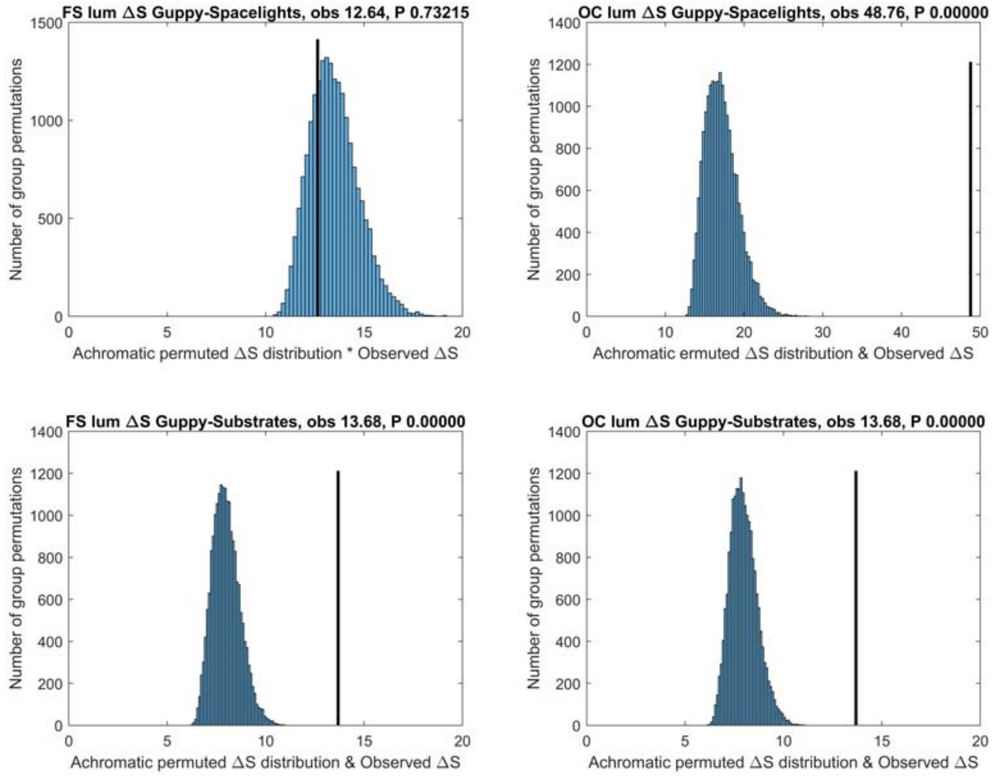

Figure S16. Permutation tests on luminance comparing Guppy spots and either spacelight or substrates, separately under FS (Forest Shade) or OC (Cloudy). Note different scale for Guppy-Spacelight OC. Same symbolism as Fig. S12.

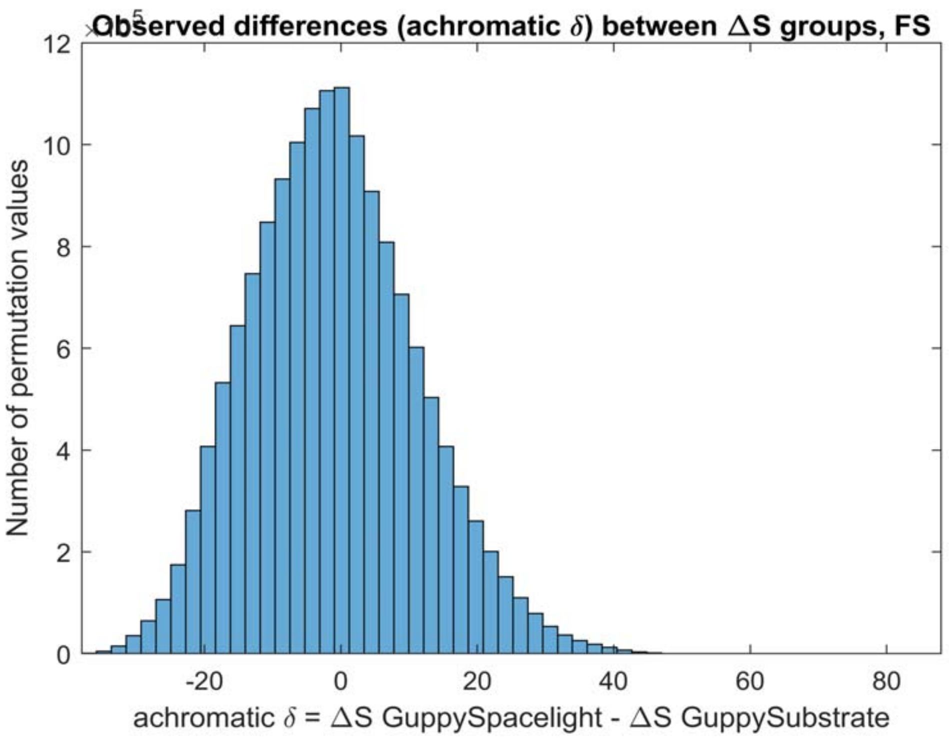

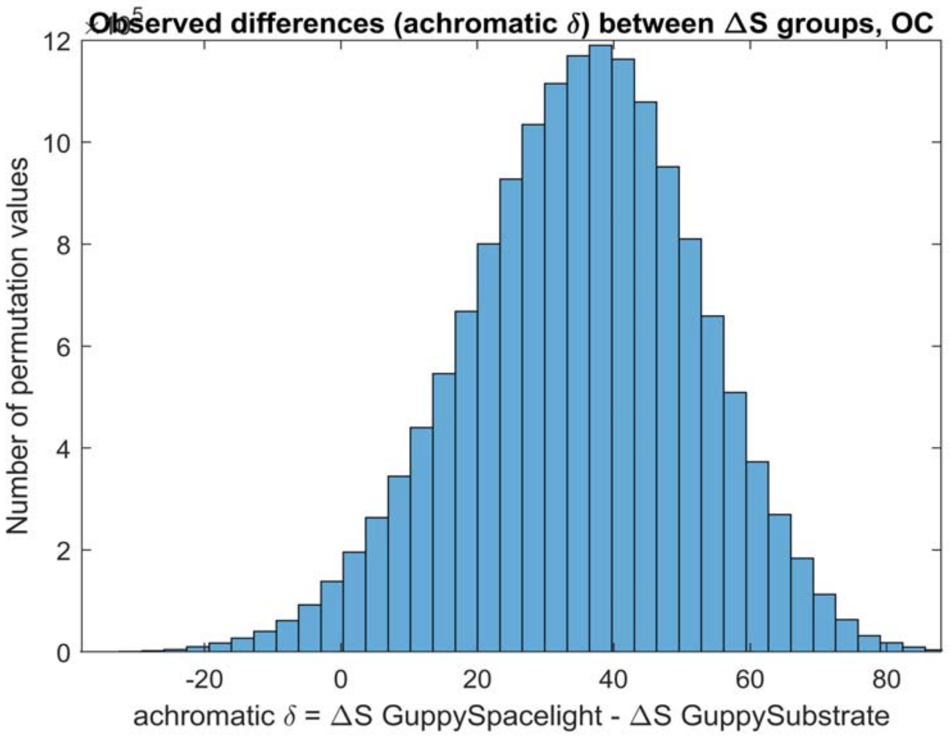

Figure S17. Distributions of differences (δ) in achromatic ΔS: $\delta = \Delta S_{GS} - \Delta S_{GB}$, where ΔS_{GS} is measured between guppy and spacelight and ΔS_{GB} is measured between guppies and substrate backgrounds. Positive values indicate that guppies are more contrasting against spacelights than substrates, and the opposite for negative values.

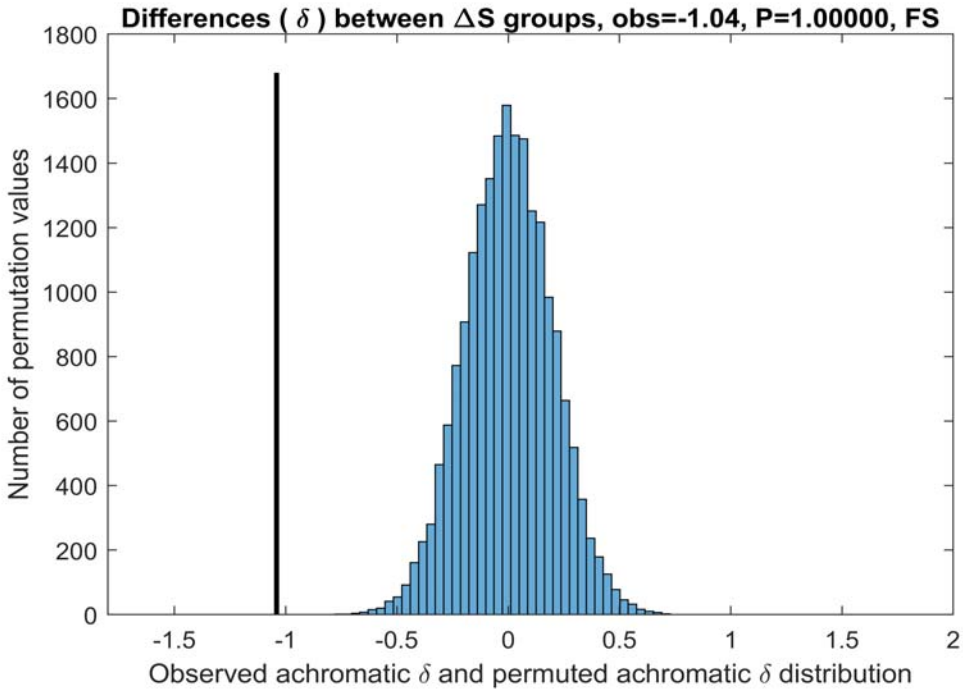

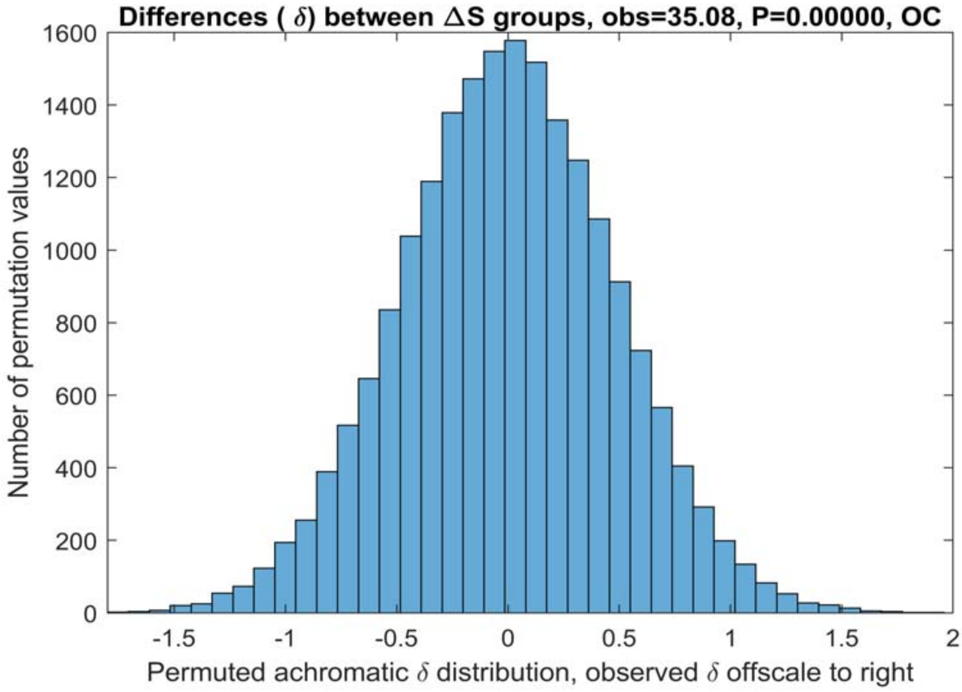

Figure S18. Results of permutation tests of the differences (δ) in achromatic ΔS measured by $\delta = \Delta S_{GS} - \Delta S_{GB}$, where ΔS_{GS} is measured between guppy and spacelights and ΔS_{GB} is measured between guppies and substrate backgrounds. Symbolism as in fig. S14. ***The observed mean absolute difference is not shown here for OC because it is well beyond the right margin;*** observed values are -1.04 and 35.08 for FS and OC, respectively. If $\delta < 1$ the two groups are not distinguishable ($\delta = \Delta S = 1$ is a JND). In FS (Forest Shade) $\delta = -1.04$ and on average the achromatic contrast probably does not change noticeably between spacelight and substrates, although guppies might be marginally more achromatically conspicuous against substrates. However, in OC (Cloudy) conditions guppies are much more achromatically conspicuous against spacelights ($\delta = 35.08$). This pattern is very different from chromatic contrast, where there is a significant spacelight advantage under both lighting conditions ($\delta = 7.63$, 7.34, Fig. S14).

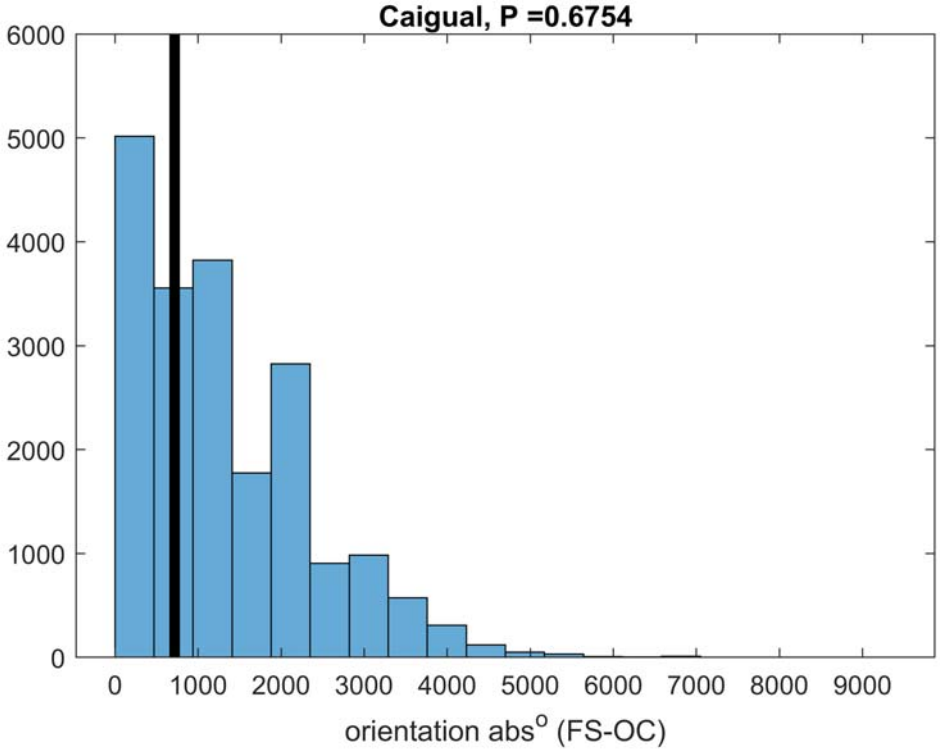

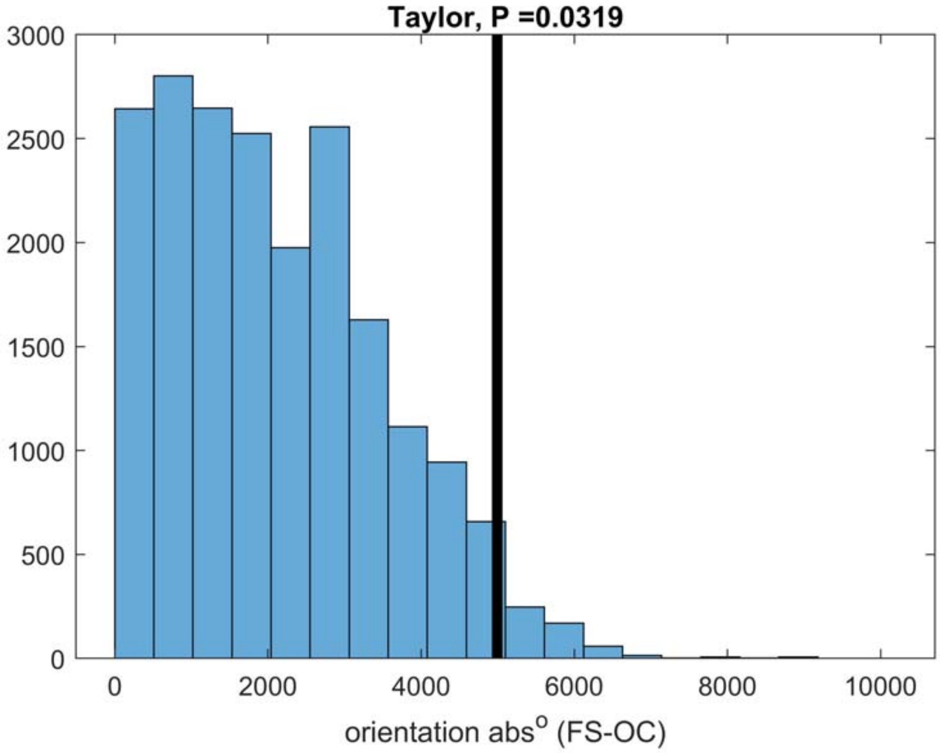

Figure S19. Permutation tests for differences in orientation between FS and OC, data shown in Fig. 5. Symbolism the same as earlier permutation tests. No evidence for differences within Caigual (P=0.67) but significant differences in Taylor (P=0.032). Fig. 5 shows that Taylor fish orient more towards the spacelight from the opposite side of the stream in OC but orient relatively more towards downstream in FS.

Table S1, Goodness of fit χ^2 tests with 1:1:1:1 expected (3df) for each row of the transition matrices in Table 3. A significant deviation ($P<0.05$) means that a pair with a given orientation (a given row of the table; orientation D1, D2, D3, or D4) does not change orientation at random.

Direction	Caigual		Taylor		Both Pooled	
code	χ^2	P	χ^2	P	χ^2	P
D1	6.12	0.1058	6.95	0.0734	4.63	0.2008
D2	12.17	0.0068	10.49	0.0148	8.03	0.0454
D3	8.11	0.0438	7.68	0.0530	12.46	0.0060
D4	14.09	0.0028	18.45	0.0004	31.26	<0.0001
